# Pravastatin for the prevention of recurrent hypertensive disorders of pregnancy: study protocol for a randomized, open-label, parallel-group, three-arm trial

**DOI:** 10.1186/s13063-025-09136-7

**Published:** 2025-11-12

**Authors:** Keiichi Kumasawa, Kosuke Kashiwabara, Mariko Inoue, Kayoko Kaneko, Hironobu Hyodo, Takahiro Yamashita, Takayuki Iriyama, Takeshi Nagamatsu, Mayumi Sako, Toru Kobayashi, Naoto Takahashi, Shigeru Saito, Yasushi Hirota, Yutaka Osuga

**Affiliations:** 1 Department of Obstetrics and Gynecology, Faculty of Medicine, The University of Tokyo, 7-3-1 Hongo, Bunkyo-Ku, Tokyo, 113-8655 Japan; 2 Clinical Research Promotion Center, The University of Tokyo, Tokyo, Japan; 3 Division of Maternal Medicine, Center of Maternal-Fetal, Neonatal and Reproductive Medicine, National Center for Child Health and Development, Tokyo, Japan; 4 Department of Obstetrics and Gynecology, Tokyo Metropolitan Bokutoh Hospital, Tokyo, Japan; 5 Department of Obstetrics and Gynecology, Aiiku Hospital, Tokyo, Japan; 6 Department of Clinical Research Promotion, Clinical Research Center, National Center for Child Health and Development, Tokyo, Japan; 7 Department of Data Science, Clinical Research Center, National Center for Child Health and Development, Tokyo, Japan; 8 Department of Pediatrics, Faculty of Medicine, The University of Tokyo, Tokyo, Japan; 9 University of Toyama, Toyama, Japan; 10 Teikyo Academic Research Center, Teikyo University, Tokyo, Japan; 11 Department of Obstetrics and Gynecology, Teikyo University School of Medicine, Tokyo, Japan

**Keywords:** Efficacy, Hypertensive disorders of pregnancy, Outcomes, Pravastatin, Randomized trial, Safety, Study protocol

## Abstract

**Background:**

Hypertensive disorders of pregnancy lead to a critically ill maternal/fetal state due to maternal organ failure and fetal dysfunction related to placental abruption or impaired placental circulation. No treatment with established efficacy for hypertensive disorders of pregnancy is currently available, and delivery is the only definitive treatment. This article describes the study protocol for a trial that aims to evaluate whether early pravastatin treatment is effective in preventing hypertensive disorders of pregnancy.

**Methods:**

A randomized, open-label, parallel-group, three-arm trial will be conducted with aspirin administered as the baseline treatment across all groups. Pregnant women with a history of hypertensive disorders of pregnancy who are at high risk of developing hypertensive disorders of pregnancy will be recruited from four recruitment sites in Japan. The three interventions of the study comprise oral pravastatin 5 mg once daily with oral bayaspirin 100 mg once daily, oral pravastatin 10 mg once daily with oral bayaspirin 100 mg once daily, and oral bayaspirin 100 mg once daily alone. The primary outcome measure is the incidence of hypertension during pregnancy. The secondary outcome measures include incidence rates of preeclampsia and gestational hypertension, maternal serum soluble fms-like tyrosine kinase-1/placental growth factor ratio, as well as soluble fms-like tyrosine kinase-1 and placental growth factor levels, whether the mother is proteinuric, placental weight, umbilical cord blood lipid profile, the incidence of hypertensive disorders of pregnancy-related complications (placental abruption, hemolysis, elevated liver enzymes, and low platelet count syndrome, and eclampsia), week at diagnosis of hypertensive disorders of pregnancy, incidence of severe hypertensive disorders of pregnancy, and neonatal outcomes (birth weight, percentage of small for gestational age neonates, neonatal intensive care unit admission rate, and auditory brainstem response).

**Discussion:**

The results of this study are expected to have wide-ranging implications given the effects of hypertensive disorders of pregnancy on long-term maternal and child health, as well as health economics.

**Trial registration:**

Japan Registry of Clinical Trials (Trial ID: jRCTs031230067; registered May 11, 2023; https://jrct.mhlw.go.jp/en-latest-detail/jRCTs031230067).

**Supplementary Information:**

The online version contains supplementary material available at 10.1186/s13063-025-09136-7.

## Background

### Background and rationale

Pregnancy-related high blood pressure is known as hypertensive disorders of pregnancy (HDP). HDP is progressive and can critically affect both the mother and fetus, leading to maternal organ failure and placental dysfunction (e.g., placental abruption or impaired placental circulation). Because the progression of HDP is sometimes rapid, the completion of pregnancy, that is, parturition, is the only radical solution.

HDP is a broad concept extending beyond the definition of preeclampsia. The definition of HDP in Japan was established by the Japan Society for the Study of Hypertension in Pregnancy (JSSHP) in 2018. HDP is classified into four: i) preeclampsia (PE), ii) gestational hypertension (GH), iii) superimposed preeclampsia, and iv) chronic hypertension (CH) [1].

HDP reportedly occurs in approximately 10% of all pregnancies [2]. HDP is complicated by serious perinatal conditions that are critical and life-threatening for mothers, including eclampsia; central nervous system disorders; hemolysis, elevated liver enzymes, low platelet count (HELLP) syndrome; pulmonary edema; perinatal myocarditis; placental abruption; and cerebral hemorrhage.

HDP has significant effects on maternal and fetal health. Placental insufficiency associated with HDP can impair uterine-placental circulation, thereby causing fetal growth restriction.

No treatment with established efficacy for HDP is currently available, and delivery is the only definitive treatment. However, efforts to develop preventive measures against HDP or identify predictive factors for this condition have made some progress. Low-dose aspirin (LDA) has been investigated for PE prophylaxis since 1979. In 2017, Roknik et al. administered either LDA or a placebo to algorithm-identified pregnant women at a high risk of developing PE. They reported that the incidence of PE by gestational week 37 was 1.6% (13/798) in the LDA group compared with 4.3% (35/822) in the placebo group (odds ratio [OR]: 0.38) [3]. The overall incidence of PE across the entire pregnancy duration was 8.3% (66/798) in the LDA group compared with 11.4% (94/822) in the placebo group [3]. This means that a substantial number of pregnant women treated with LDA developed PE and that oral LDA was not effective in preventing PE. Therefore, no effective preventive measures against HDP including PE are currently available.

Statins, inhibitors of 3-hydroxy-3-methylglutaryl-coenzyme-A (HMG-CoA) reductase, have been shown to be effective in the primary and secondary prevention of cardiovascular mortality and morbidity in multiple studies [4, 5]. There are notable overlaps in pathophysiological features between PE and cardiovascular disease in adults, particularly relating to inflammation and endothelial injury [6–8]. Furthermore, statins have been utilized in animal models of PE to address angiogenic imbalance, one of the pathological hallmarks of PE, and restore endothelial dysfunction [7, 9, 10]. In our previous study using a PE model mouse, we demonstrated that pravastatin upregulates placental growth factor (PlGF) and downregulates soluble fms-like tyrosine kinase-1 (sFlt-1), leading to improvement in the PE phenotype [11]. These changes indicate an improvement in placental function, considered a key mechanism through which pravastatin may ameliorate PE. The resulting plausibility from a biological perspective, in combination with data from animal studies [4, 9], support a potential role for statin administration in the prevention of PE. Notably, pravastatin does not directly reduce blood pressure; instead, it ameliorates placental dysfunction by modulating angiogenic factors such as those described above. There is also in vitro evidence that this benefit occurs even at low doses of pravastatin [12].

Of all the statins available, pravastatin has been the most widely studied in this context [13]. While six statins are currently marketed, they have different strengths in relation to their ability to reduce LDL cholesterol levels and different solubility properties (hydrophilic and lipophilic). If statins are to be used during pregnancy, a standard statin is preferable to a strong statin, to avoid excessive cholesterol reduction. Hydrophilic statins are less dependent on hepatic metabolism and are therefore considered safer for patients with impaired liver function. Additionally, since PE is often associated with hepatic dysfunction, a hydrophilic statin that can be used safely in such conditions is desirable. Pravastatin meets both these criteria, being a standard statin with mild cholesterol-lowering effects and a hydrophilic profile that allows its use in patients with hepatic impairment. Moreover, lipophilic statins tend to cross the placenta more readily, raising concerns about fetal exposure. Therefore, it is preferable to avoid lipophilic statins and to select a hydrophilic statin such as pravastatin.

Ascertaining human data on the safety of pravastatin to prevent PE has been limited in part due to the challenges of studying drugs in pregnancy for off-label indications [14]. A meta-analysis of nine studies that evaluated pregnancy and childbirth outcomes associated with statin exposure during pregnancy found statin use to be associated with a significant increase in spontaneous abortion rates (*n* = 8422 women included), as well as a non-significant trend for increased stillbirth rate (*n* = 2,350), preterm delivery (*n* = 483), and both induced and elective abortion rates (*n* = 8422) [15]. Notably, these findings may be influenced by confounding factors. In particular, there are no robust data comparing miscarriage rates between women who required statin therapy due to underlying maternal conditions but did not take statins and those who did. Therefore, it remains unclear whether the observed increase in adverse pregnancy outcomes is attributable to statin exposure itself or to the underlying maternal comorbidities necessitating statin use. Furthermore, this is a highly complex area, and these data must be balanced against the risk of PE to mother and fetus. Another systematic review and meta-analysis of 14 studies including 1570 pregnant women receiving pravastatin or placebo for PE prevention found supportive evidence for pravastatin use in terms of reduced numbers of neonates born with intrauterine growth restriction (*n* = 277), neonatal admissions to intensive care (*n* = 180), and the incidence of preterm deliveries (*n* = 293) [13].

Nevertheless, recent years have seen a growing accumulation of clinical and preclinical data supporting the safety of pravastatin use during pregnancy in terms of teratogenicity [9, 16]. Retrospective studies and case reports have demonstrated that inadvertent exposure to pravastatin in early pregnancy does not appear to increase the risk of congenital anomalies [17, 18].

These findings collectively suggest that pravastatin may be safer in pregnancy than previously assumed and have contributed to the U.S. FDA’s decision in 2021 to lift the formal contraindication against statin use in pregnancy, particularly for women with high cardiovascular risk. This decision reflects growing recognition that, in selected high-risk populations—such as women with severe hypercholesterolemia or high cardiovascular risk—the potential benefits of statin therapy may outweigh the theoretical risks, especially when using hydrophilic agents like pravastatin that have lower transplacental transfer. Nevertheless, further data are urgently needed on the safety and efficacy of pravastatin use in the prevention of PE.

Regarding predictive markers for HDP, a high sFlt-1/PlGF ratio in the maternal blood is useful for predicting the occurrence of HDP, and laboratory tests for assessing this parameter are becoming popular in Japan. However, this marker has not been used effectively to prevent and treat HDP.

### Objectives

This study aims to test the hypothesis that pravastatin treatment during early pregnancy is safe and effective in preventing HDP [11]. All participants will receive aspirin, and only those assigned to the test group will receive pravastatin in addition to aspirin. The incidence of HDP will be compared between the pravastatin and control groups (that received aspirin only). We decided that administering low-dose aspirin to all participants, including those in the control group, was ethically appropriate and consistent with best clinical practice. Although the efficacy of aspirin in preventing hypertensive disorders of pregnancy is limited, it is thought to provide some preventive benefit. Therefore, we considered background use of aspirin necessary to ensure that all participants received the current standard of care.

Several studies outside of Japan [19–21] that assessed the efficacy of pravastatin at 10, 20, and 40 mg/day have demonstrated the non-inferiority of 10 mg/day over higher doses. Considering this and the differences in body weight between Japanese and non-Japanese women, as well as the aforementioned evidence supporting that pravastatin appears to be effective at improving angiogenic imbalance even at low doses [12], pravastatin will be administered at 5 or 10 mg/day in this study. Lower drug doses are associated with lower risks of adverse reactions.

In this study, pravastatin and aspirin will be administered to pregnant women with a history of HDP who are at a high risk of developing HDP for an unapproved indication (in an off-label manner). Therefore, this study will be specified as clinical research, as defined in Item 2, Article 2 of the Clinical Trials Act, and will be conducted in compliance with the Clinical Trial Standards specified in Article 3 of the Act.

This study is designed to evaluate the safety and efficacy of orally administered pravastatin for preventing HDP in pregnant women at high risk of developing HDP (high-risk pregnant women) who have a history of HDP. For this purpose, the incidence of HDP will be compared between patients treated with pravastatin (5 or 10 mg/day) and those not treated with the drug.

The trial protocol has been reported in line with the SPIRIT guidelines [22] and the accompanying checklist has been provided in the Additional File 1: SPIRIT Checklist.

## Methods/Design

### Trial design

**Fig. 1:**
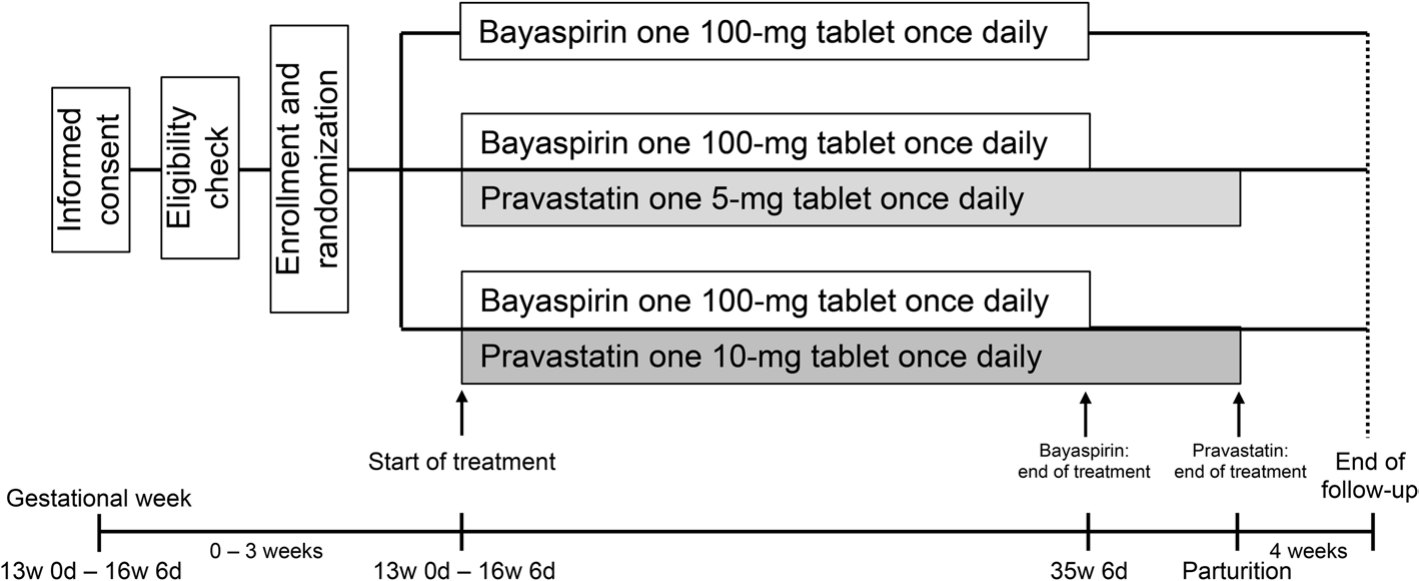
An outline of the study design

This will be a randomized, open-label, parallel-group trial with aspirin administered as a baseline treatment across all groups. Three treatment arms will be used, as shown in Fig. 1.

### Study setting and recruitment

Four recruitment sites are planned, as shown in Table 1. Detailed information about the recruitment plan is provided in the Additional File 2: Supplementary Methods.

**Table 1:** Participating institutions and planned sample sizes

Participating institutions	Planned sample size
The University of Tokyo Hospital	*n* = 30
Aiiku Hospital	*n* = 10
National Center for Child Health and Development	*n* = 30
Tokyo Metropolitan Bokutoh Hospital	*n* = 20

### Inclusion criteria

Those who meet all the following requirements will be considered for study enrolment: having a history of HDP during any previous pregnancy, gestational age between 13 weeks + 0 day and 16 weeks + 6 days after obtaining informed consent, chronological age of 18–45 years at informed consent, female sex, having provided written informed consent for the study of her own prior to enrolment, and ability to make outpatient visits as scheduled for the study.

### Exclusion criteria

Those who have any of the following conditions will be excluded from the study: APS diagnosis; chronic hypertension; multiple pregnancy; severe obesity, defined as a BMI ≥ 30 kg/m^2^ at the point of informed consent; a history of allergy to aspirin, any salicylate, or pravastatin; history of serious drug allergy (including anaphylactic shock) or any serious adverse drug reactions; participation in any clinical research (including clinical trials) and receipt of either study drug within 17 weeks before providing informed consent; presence or history of any serious hepatic disorder; alcoholism; stage ≥ 4 chronic kidney disease; current treatment with any fibrate, immunosuppressant, or nicotinate; untreated hypothyroidism (excluding latent hypothyroidism); any hereditary muscle disease or a family history thereof; a history of drug-induced muscle disorder; peptic ulcer; bleeding diathesis (excluding an aspirin-induced one); aspirin-induced asthma or a history thereof; and any other condition that disqualifies the patient from the study in the opinion of the investigator or any designated sub-investigator.

### Consent

The investigator or any designated sub-investigator will obtain written informed consent from a candidate participant and then determine whether she meets all inclusion criteria and does not meet any of the conditions included in the exclusion criteria.

### Interventions

As indicated in Fig. 1, the three interventions in the study are oral pravastatin 5 mg once daily with oral bayaspirin 100 mg once daily, oral pravastatin 10 mg once daily with oral bayaspirin 100 mg once daily, and oral aspirin 100 mg once daily alone. Bayaspirin will be used as the ISSHP Guideline [23] recommends that pregnant women who have any established strong clinical risk factor for PE should preferably start receiving low-dose aspirin therapy before gestational week 16. Pravastatin and bayaspirin will be sourced from Sawai Pharmaceutical Co., Ltd. (Osaka, Japan) and Bayer Pharma Japan (Tokyo, Japan), respectively.

For those receiving pravastatin, oral treatment will be started at visit 2 between gestational 13 weeks + 0 days and 16 weeks + 6 days and will continue until the day of parturition. The treatment may be discontinued prematurely at the discretion of the investigator if the need for any emergency procedure or operation is indicated. The participants should not breastfeed for 6 h after the last dose of the drug.

Regarding Bayaspirin, oral treatment will be started at visit 2 between gestational 13 weeks + 0 days and 16 weeks + 6 days and will be continued until gestational 35 weeks + 6 days. Those who will receive oral treatment with bayaspirin will be switched to the study treatment at visit 2 (or on the following day if the participant has taken the dose of bayaspirin scheduled on the day of the visit).

If a hemorrhage or hematoma is found, oral treatment with bayaspirin will be suspended and reinstated after the amelioration of the hemorrhage/hematoma at the discretion of the investigator. If an emergency cesarean section is anticipated, oral treatment with bayaspirin will be discontinued at the discretion of the investigator. Monitoring procedures are in place to assess and evaluate adherence to the intervention protocols, including regular follow-ups and medication logs.

### Randomization and assignment of interventions

The treatment assigned to each participant will be displayed on the electronic data capture system (CubeCDMS, Seoul, Korea) once the necessary information on the participant (including randomization factors) has been entered and confirmed. The investigator or any designated sub-investigator will start providing the study treatment assigned to each participant. Upon enrollment, a unique identification code specific to the study will be issued automatically to each participant. The investigator/sub-investigator will create a cross-reference table with the participant identification and assigned treatment and securely keep the table. Randomization will be performed through the minimization method [24] with the following randomization factors: age (≥ 40 or < 40 years) and history of PE (yes or no). All participating centers are comprehensive perinatal care centers that manage the most severe cases; therefore, stratification by center was not performed.

### Criteria for termination of participation

A participant will be withdrawn from the study if she develops hypertension at a gestational age < 20 weeks, withdraws consent to the study of her own free will, is unable to continue participating in the study in the opinion of the investigator/sub-investigator because of worsening of the underlying condition, any concomitant condition, or occurrence of any serious adverse event, is found to have any significant non-compliance with the Clinical Trials Act or its Enforcement Regulation or any significant deviation from the protocol (such as failure to meet the inclusion criteria or presence of any condition included in the exclusion criteria), or becomes unable to comply with the protocol, as well as if the entire study has been terminated, or the participant is unable to continue participating in the study in the opinion of the investigator/sub-investigator for any other reason.

When terminating a subject’s participation in the study based on the above criteria, the investigator/sub-investigator will ensure appropriate management of the subject and will clearly state the date/time of and reason for termination as well as the clinical course after termination in the subject’s medical record. All deviations from the trial protocol will be fully documented in a breach report form. Furthermore, all clinically significant abnormalities (including abnormal laboratory findings) will be examined appropriately and followed up until the abnormalities return to the medically acceptable range or further follow-up is no longer needed in the opinion of the investigator/sub-investigator. Every adverse event that is ongoing at study termination will be followed up until the event has completely resolved or further follow-up is no longer needed in the opinion of the investigator/sub-investigator. Subjects will be followed up within the framework of the study for three months; thereafter, any issues will be managed as part of routine clinical care. Those subjects who wish to withdraw consent to the study after starting to receive the study treatment will be discontinued from the study, regardless of the reason for consent withdrawal. After such study discontinuation, the investigator/sub-investigator will make efforts to determine, whenever possible, whether the consent withdrawal is related to the lack of efficacy of drug(s), any adverse event, or any incidental event (e.g., move to a new address). In any case, the investigator/sub-investigator will respect the subject’s will and take care to prevent any adverse effect due to the reason for the withdrawal of consent.

### Data collection

The following demographic and baseline characteristics will be collected from the participants: year/month of birth, age, race, height, smoking status (current, never, or ex-smoker; number of cigarettes smoked; number of years smoking), concomitant illnesses, previous illnesses, currently used oral drugs (name of drugs and duration of treatment), obstetric history (number of pregnancies, age at pregnancy, number of parturitions, gestational weeks at parturition, live or stillbirths, number of spontaneous abortions, gestational weeks at abortion, excluding the current pregnancy), use of assisted reproductive technology for the current pregnancy, and gestational age at informed consent.

**Fig. 2:**
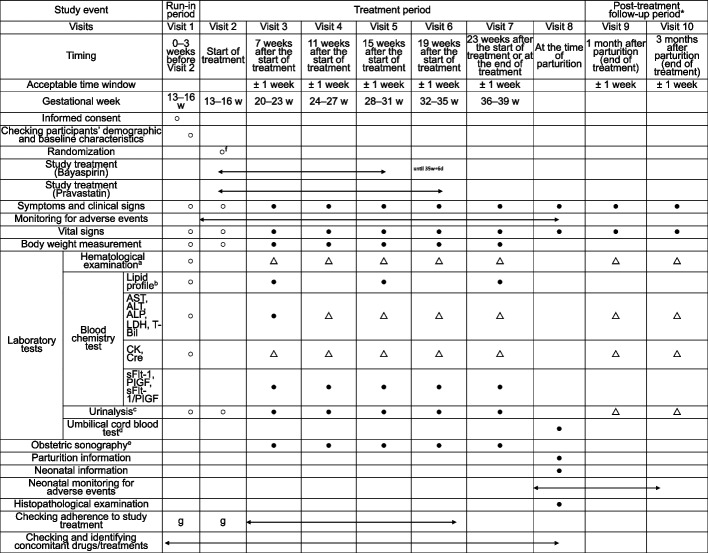
Study schedule

An outline of the study schedule is provided in Fig. 2, and information about the blood tests to be performed on participants is provided in Table 2. A full overview of data to be collected during the participant visits is provided in the Additional File 2, Supplementary Methods.

**Table 2:** Laboratory tests to be performed on study participants

Modality	Specific tests performed
Hematological examination	White blood cell count, differential neutrophil count (%), differential lymphocyte count (%), red blood cell count, hemoglobin concentration, hematocrit, and platelet count
Blood chemistry test	AST, ALT, ALP, LDH, T-Bil, CK, creatinine, T-Chol, TG, LDL-C, HDL-C, sFlt-1, PIGF, and sFlt-1/PlGF
Urinalysis	Quantitative determination of protein in urine (protein/creatinine ratio [mg/mg × Cre] using a casual urine sample) or qualitative test for proteinuria
Umbilical cord blood test	sFlt-1, PlGF, sFlt-1/PlGF, T-Chol, TG, LDL-C, HDL-C, AST, ALT, CK, NSE, TSH, FSH, LH, and E2

*AST* aspartate transferase, *ALT* alanine transferase, *ALP* alkaline phosphatase, *LDH* lactate dehydrogenase, *T-Bil* total bilirubin, *CK* creatine kinase, *T-Chol* total cholesterol, *TG* triglycerides, *LDL-C* low density lipoprotein-cholesterol, *HDL-C* high density lipoprotein cholesterol, *sFlt-1* soluble fms-like tyrosine kinase-1, *PlGF* placental growth factor, *NSE* neuron-specific enolase, *TSH* thyroid-stimulating hormone, *FSH* follicle-stimulating hormone, *LH* luteinizing hormone, *E2* estradiol

### Primary outcomes

The primary outcome measure will be HDP incidence. The cumulative and each visit incidence of HDP (frequency/percentage) will be compared between treatment groups.

A participant will be regarded as developing HDP if she meets the diagnostic criteria for i) PE or ii) GH, that is, the major categories of HDP specified in the 2018 JSOG Terminology, Definition, and Classification of Hypertensive Disorders of Pregnancy.

The primary analysis will compare the combined pravastatin groups (5 mg + 10 mg) with the control group, while exploratory analyses will include comparisons between the 5 mg group and the control group, the 10 mg group and the control group, and the 5 mg and 10 mg groups themselves.

### Secondary outcome measures

The secondary outcome measures of the study will be as follows: incidence rates of PE and GH (two major categories of HDP), maternal serum sFlt-1/PlGF ratio and sFlt-1 and PlGF levels, whether the mother is proteinuric, placental weight, umbilical cord blood lipid profile, incidence of HDP-related complications (placental abruption, HELLP syndrome, and eclampsia), week at diagnosis of HDP, incidence of severe HDP, and neonatal outcomes (birth weight, percentage of SGA neonates, neonatal intensive care unit admission rate, and auditory brainstem response).

As specified in the 2018 JSOG Terminology, Definition, and Classification of Hypertensive Disorders of Pregnancy, HDP will be graded as “severe” if either of the following criteria are met: (1) PE or GH with systolic blood pressure ≥ 160 mmHg or diastolic blood pressure ≥ 110 mmHg, or (2) PE complicated by maternal organ disorder or uterine placental insufficiency.

### Safety outcome measures

The following safety outcome measures will be evaluated: (1) frequency/percentage of participants experiencing adverse events, and (2) frequency and percentage of abortions and stillbirths per pregnancy.

An adverse event will be judged as serious if it results in death, is life-threatening, requires hospitalization or prolonged hospitalization for therapeutic purposes, results in disability, may result in disabilities, is as serious as the aforementioned outcomes, or is a congenital anomaly or congenital disability.

Hospitalization scheduled before study participation or hospitalization/prolonged hospitalization for diagnostic purposes is not considered serious. Fetal or neonatal death will be regarded as meeting the seriousness criterion [1].

### Statistical analysis

#### Sample size calculation

A total of 90 participants will be randomized to receive pravastatin at 10 mg/day (*n* = 30), 5 mg/day (*n* = 30), or no pravastatin (*n* = 30). Based on the results of previous studies (see “Sample size and recruitment” section of the Additional File 2: Supplementary Methods for further information), the incidence of HDP in this study is expected to be 10% in the pravastatin 10 mg/day group and 50% in the non-treated group. Assuming that the significance level for the entire study is 5% on a two-tailed basis and that Fisher’s least significant difference test (Fisher’s exact test for three-group and two-group comparisons) will be used, a sample size of *n* = 27 per group would provide sufficient statistical power to show the superiority of active treatment (pravastatin 10 mg/day and/or 5 mg/day) over non-treatment, based on various hypotheses concerning the incidence of HDP (Table 3).

**Table 3:** Statistical power analysis of different pravastatin doses for preventing hypertensive disorders of pregnancy

	Hypothetical incidence of HDP (%)			Statistical power (%)^a^			
	Pravastatin 10 mg/day group	Pravastatin 5 mg/day group	Non-treated group	Superiority of pravastatin at 10 mg/day	Superiority of pravastatin at 5 mg/day	Superiority of pravastatin at either dose level	Superiority of pravastatin at both dose levels
Hypothesis 1	10	10	50	86.7	87.0	93.9	79.8
Hypothesis 2	10	10	40	62.3	61.7	74.7	49.3
Hypothesis 3	10	20	50	84.8	53.8	86.7	51.9
Hypothesis 4	10	20	40	59.3	26.1	61.7	26.1

*HDP* hypertensive disorders of pregnancy

^a^ Superiority of one over another is defined as follows:

Superiority of pravastatin at 10 mg/day: A significantly lower incidence of HDP in the pravastatin 10 mg/day group than in the non-treated group

Superiority of pravastatin at 5 mg/day: A significantly lower incidence of HDP in the pravastatin 5 mg/day group than in the non-treated group

Superiority of pravastatin at either dose level: Significantly lower incidence of HDP in the pravastatin 10 mg/day or 5 mg/day groups than in the non-treated group

Superiority of pravastatin at both dose levels: Both the pravastatin 10 mg/day and 5 mg/day groups showed a significantly lower incidence of HDP compared to the non-treated group

Missing outcomes and cross-over are expected to be minimal due to the short follow-up period and strict eligibility criteria. However, should such cases occur, we plan to address them through appropriate statistical approaches, such as sensitivity analyses or multiple imputation for missing data. Given the rarity of eligible patients and the high-risk nature of the target population, increasing the sample size further would present significant recruitment challenges. Therefore, we believe the current sample size is reasonable and sufficient to achieve the study objectives.

#### Analysis sets

A number of analysis sets will be defined by the study (see Additional File 2: Supplementary Methods), but the main analyses for the primary endpoint will be performed on the “Full Analysis Set (FAS)” comprising participants enrolled in this study and treated with at least one dose of the study drug(s) after randomization and who had no major protocol violations (such as not providing informed consent and being enrolled outside the period of enrolment). “Full Analysis Set 2 (FAS2),” comprising participants who did not develop hypertension before gestational week 20, will be used as the primary population for analysis of the secondary endpoints.

#### Variables and analysis plan

All efficacy endpoints will be analyzed based on the intent-to-treat principle, and comparisons will be made between groups based on the assigned treatments. More detailed information about the statistical analysis plan is provided in the Additional File 2, Supplementary Methods, and the full statistical analysis plan is provided as a separate Additional File 3, Statistical Analysis Plan. No interim analyses have been planned for the study, because of (i) the ethical implications of conducting an interventional trial on pregnant participants and the need to minimize burden on them and (ii) the relatively small planned sample size of the study, meaning that an interim analysis would likely lack sufficient statistical power to meaningfully assess efficacy or futility. Furthermore, performing such an interim analysis could increase the risk of a type I statistical error without appropriate statistical adjustments, complicating the interpretation of the final results.

Summary statistics will be generated for all relevant variables, including participant demographic and baseline variables. Between-group comparisons of the demographic and baseline characteristics will be performed using the chi-square test for nominal variables and one-way analysis of variance or the Kruskal–Wallis test for continuous variables.

Regarding the primary endpoint analysis, for the incidence of HDP at each specified time point, frequencies, percentages, and a two-tailed 95% confidence interval (CI) for the percentages will be calculated. Between-group comparisons will be made, with correction for multiple comparisons, using Fisher’s least significant difference test. For the secondary endpoints, no adjustments will be made for multiple comparisons. Further detailed information on the statistical tests used for each of the planned analyses has been provided in the Additional File 2: Supplementary Methods.

Data will be considered significant when the two-tailed *p*-value is < 0.05.

### Oversight and monitoring

#### Monitoring

For the quality control of clinical research, the Principal Investigator will prepare written procedures for monitoring this study and submit them together with this protocol to the University of Tokyo Clinical Research Review Board for review and approval. The Principal Investigator will appoint the monitor(s) responsible for monitoring the study. According to the written monitoring procedures, the monitor(s) will check whether this study is conducted in compliance with the current version of the protocol and regulatory requirements (such as the Clinical Trials Act and its Enforcement Regulation) throughout the study and will report the results of monitoring to the investigator at the institution. After being informed of the monitoring results, the investigator notifies the Principal Investigator of the reported results, as necessary. The monitor(s) must keep the personal information of the participants confidential and available during monitoring.

#### Auditing

The Principal Investigator will direct an individual who is independent of departments responsible for performing duties for the study (including its monitoring) to audit the study to assure that this study is conducted and data from the study are prepared, recorded, and reported in compliance with all applicable regulatory requirements (e.g., Clinical Trials Act and its Enforcement Regulation), this protocol, and all written procedures for conducting clinical studies.

Regarding on-site monitoring, case-related matters will be monitored during the study period at each site, based on the following schedule (9 times per site):

1. After the first subject completes Visit 1 and before Visit 2. If this timing is not feasible, monitoring should be conducted as early as possible
2. After the first subject completes Visit 4
3. After the first subject completes Visit 8
4. After the fifth subject completes Visit 8
5. After the tenth subject completes Visit 8
6. After the fifteenth subject completes Visit 8
7. After the twentieth subject completes Visit 8
8. After the twenty-fifth subject completes Visit 8
9. After all subjects have completed all scheduled visits

Monitoring of procedural matters will be conducted annually during the study period, in conjunction with case monitoring.

After the initial on-site monitoring has been conducted, off-site monitoring will, in principle, be performed monthly based on the data entered into the Electronic Data Capture system.

#### Data and safety monitoring board

The data and safety monitoring board comprises those not directly involved in the study. When consulted by the Principal Investigator regarding any of the matters described below, the committee will discuss the matter and present its judgment.

- Whether to continue, suspend, or terminate the study based on the overall safety and efficacy information.
- Whether any serious adverse event reported is causally related to the study and how to handle the event
- Whether to amend any important aspect of the protocol relevant to the safety or efficacy of study drug(s)
- Other matters inquired by the Principal Investigator Further details of the procedures for this committee are provided in written procedures prepared separately.

#### Criteria for study termination

The Principal Investigator will determine whether to continue the study in the following situations:

1. Receipt of new information regarding the quality, efficacy, or safety of the study drug(s), or any other important findings that may affect the scientific validity or feasibility of continuing the study.
2. Difficulty in achieving the planned sample size due to challenges in subject accrual.
3. Inability to comply with instructions from the University of Tokyo Clinical Research Review Board regarding protocol amendments.
4. A decision by the University of Tokyo Clinical Research Review Board to terminate the study.
5. Identification of any significant or persistent non-compliance with the Clinical Trials Act, its Enforcement Regulation, or this study protocol.

#### Patient and public involvement

There was no involvement of patients or the public in the design, conduct, or dissemination plans of this study.

## Discussion

This article describes the protocol for a randomized controlled trial testing the hypothesis that pravastatin treatment initiated in early pregnancy is safe and effective in preventing HDP. This study addresses a major unmet clinical need: currently, no treatment with established efficacy is available for HDP, with delivery being the only definitive treatment. Accumulating data has indicated the potential utility of pravastatin in the prevention of PE, but further data are needed from well-designed clinical trials, particularly for appropriate safety data to confidently recommend this treatment option in the clinic.

Compared with previous studies on this topic, the study protocol described in this manuscript evaluates a low dose of pravastatin at 10 mg/day, as well as an even smaller dose of 5 mg/day. Demonstrating the efficacy at such low doses is highly important, considering the safety considerations for the administration of this drug to pregnant women. This study will avoid the organogenesis period in early pregnancy and begin administration between 13 and 16 weeks of gestation, considering the changes during placental formation in PE. The study involves regular measurement of maternal serum levels of sFlt-1, PlGF, and the sFlt-1/PlGF ratio throughout pregnancy, allowing evaluation of not only the onset and absence of HDP but also of the pre-disease state.

The main limitations of this study identified to date include the restriction of trial recruitment to a single country (Japan), despite being multi-center, and the relatively small target sample size. Although the study is limited to a Japanese population, the sample size has been appropriately calculated and is considered sufficient to address the study objectives. While the findings may primarily apply to Japanese individuals, this is not considered a major limitation. For example, previous studies on predictive biomarkers such as the sFlt-1/PlGF ratio have shown no significant differences between European and Asian populations [25, 26], suggesting that the biological mechanisms underlying hypertensive disorders of pregnancy are largely consistent across regions.

## Supplementary Information

Additional file 1. SPIRIT Checklist. Recommended items to address in a clinical trial protocol and related documents.

Additional file 2. Supplementary Methods. Sample size and recruitment, Statistical methods, and Data to be collected during visits.

Additional file 3. Statistical Analysis Plan. Statistical analysis plan (SAP) version 1.0.

## Data Availability

This is a protocol paper, and data collection is currently ongoing. No datasets are available at this time. The data will be shared upon reasonable request after the completion of the study.

## Abbreviations

CH: Chronic hypertension
GH: Gestational hypertension
HDP: Hypertensive disorders of pregnancy
HELLP: Hemolysis, elevated liver enzymes, low platelet count
JSSHP: Japan Society for the Study of Hypertension in Pregnancy
LDA: Low-dose aspirin
PE: Preeclampsia
