# Supplementary material for: Pravastatin for the prevention of recurrent hypertensive disorders of pregnancy: study protocol for a randomized, open-label, parallel-group, three-arm trial
Source: Trials. 2025 Nov 12;26:499. doi: 10.1186/s13063-025-09136-7 (PMC12613540; doi:10.1186/s13063-025-09136-7)
Supplement: Supplementary file 2 — Additional file 2. Supplementary Methods. Sample size and recruitment, Statistical methods, and Data to be collected during visits. [file 13063_2025_9136_MOESM2_ESM.docx]

**Additional File 2**

**Supplementary Methods**

**Sample size and recruitment**

A total of 90 participants will be randomized to receive pravastatin at 10 mg/day (n=30), 5 mg/day (n=30), or no pravastatin (n=30). The primary hypothesis to be tested in this study is that the study drug (pravastatin) at 10 mg/day or 5 mg/day is superior to no treatment in preventing hypertensive disorders of pregnancy (HDP) in pregnant women at high risk of developing HDP who have a history of HDP. Previous studies evaluating this indication for pravastatin have reported the following results: in 2016, Constantine et al. reported that among pregnant women at high risk for developing preeclampsia (PE), PE occurred in four of 10 treated with placebo compared with nil in those treated with pravastatin at 10 mg/day [1]. In 2021, Constantine et al. reported that PE occurred in five of 10 that were treated with a placebo compared with two out of 10 of those treated with pravastatin at 20 mg/day [2]. Based on these results, the incidence of HDP in this study is expected to be 10% in the pravastatin 10 mg/day group and 50% in the non-treated group. Assuming that the significance level for the entire study is 5% on a two-tailed basis and that Fisher’s least significant difference test (Fisher’s exact test for three-group and two-group comparisons) will be used, a sample size of n=27 per group would provide the study with various statistical powers to show the superiority of active treatment (pravastatin 10 mg/day and/or 5 mg/day) over non-treatment, based on various hypotheses concerning the incidence of HDP (Table 3).

If pravastatin is effective at either 10 or 5 mg/day for preventing HDP, its clinical use in this new indication is expected. Considering this, the sample size is set at n=27 per group, as this would provide the study with sufficient power to show the superiority of pravastatin at either dose level under both hypotheses 1 (as high efficacy as expected) and 2 (lower efficacy than expected), as shown in the above table. This sample size can also adequately power the study to show the efficacy of pravastatin at 10 mg/day under hypothesis 3 (lower efficacy than expected only at 5 mg/day) and hypothesis 4 (unexpectedly higher or lower incidence of HDP in both the pravastatin 5 mg/day and non-treated groups) (despite a slightly reduced power to show the superiority of pravastatin at 10 mg/day under hypothesis 4 compared with hypothesis 2).

Based on these considerations and an expected dropout rate of 10%, the target sample size was set at n=30 per group.

To ensure the achievement of this target sample size, a greater number of participants will be enrolled at each participating institution than the planned sample size set per institution, as shown in Table 1. The Principal Investigator will control the recruitment process so that the total number of participants enrolled in this study (cumulative number across the four institutions) will not exceed the target sample size. If it is necessary to increase the target sample size of the entire study, the Principal Investigator should receive prior approval from the University of Tokyo Clinical Research Review Board and report the changes to the Minister of Health, Labor, and Welfare.

**Statistical methods**

The following analysis sets will be defined by the study:

- Full Analysis Set (FAS): participants enrolled in this study and treated with at least one dose of the study drug(s) after randomization and who had no major protocol violations (such as not providing informed consent and being enrolled outside the period of enrolment).
- Full Analysis Set 2 (FAS2): participants who did not develop hypertension before gestational week 20.
- Per Protocol Set (PPS): FAS2 participants who are more compliant with the protocol, that is, have no significant protocol violations regarding study methodology and concomitant treatments, such as not meeting the inclusion criteria, having any of the conditions included in the exclusion criteria, and is not fully (<80%) adherent to pravastatin treatment
- Safety analysis: participants enrolled in this study and treated with at least one dose of the study drug(s).

***Statistical variables and analysis plan***

Statistical analyses will be performed after all participants have completed the study treatment, and all data will be frozen. All efficacy endpoints will be analyzed based on the intent-to-treat principle, and comparisons will be made between groups based on the assigned treatments. The FAS will be used as the primary population for analysis of the primary endpoint. FAS2 and PPS will also be analyzed for reference purposes. FAS2 will be used as the primary population to be analyzed for secondary endpoints. For reference, PPS analyses will also be performed. For the safety assessment, comparisons will be made between groups according to the actual treatments received. A safety analysis will be performed on the safety analysis set. Details of the statistical analyses are provided in the Statistical Analysis Plan.

***Analysis of participants’ demographic and baseline characteristics***

For the participants’ demographic and baseline variables, the data on the participants in each analysis set will be summarized in terms of distribution and summary statistics. For nominal variables, the frequencies and percentages of individual categories will be displayed by group. For continuous variables, summary statistics (N, mean, standard deviation, minimum, median, and maximum) will be calculated for each group. Between-group comparisons will be performed using the chi-square test for nominal variables and one-way analysis of variance or the Kruskal–Wallis test for continuous variables. Data will be considered significant when the two-tailed p-value is < 0.05.

***Primary endpoint analysis***

The primary objective of this study is to show the superiority of pravastatin (the study drug) at 10 mg/day and 5 mg/day over non-treatment in preventing HDP in pregnant women at a high risk of developing HDP who have a history of HDP.

Hence, the analysis of the primary endpoint will be based on the differences in the incidence of HDP in the pravastatin 10 mg/day and 5 mg/day groups compared with the non-treated group. In the hypothesis test for the superiority of pravastatin at 10 mg/day, the null hypothesis is that the incidence of HDP in the 10 mg/day group minus the incidence of HDP in the non-treated group is zero, whereas the alternative hypothesis is that the incidence of HDP in the 10 mg/day group minus the incidence of HDP in the non-treated group is not zero. In the hypothesis test for the superiority of pravastatin at 5 mg/day, the null hypothesis is that the incidence of HDP in the 5 mg/day group minus the incidence of HDP in the non-treated group is zero. In contrast, the alternative hypothesis is that the incidence of HDP in the 5 mg/day group minus the incidence of HDP in the non-treated group is not zero. Adjustments for multiple comparisons will be made using Fisher’s least significant difference test, which is a reasonable statistical method for three-group comparisons. It can be concluded that the superiority of active treatment is demonstrated when the point estimate of the between-group difference in the incidence of HDP is a negative value and when the incidence in the 10 mg/day or 5 mg/day groups is significantly lower than that in the non-treated group. The significance level was set at 5% using a two-tailed test.

Participants who withdraw from the study after gestational week 20, before developing HPD, and before parturition will be regarded as not developing HPD. If such participants account for more than 10% of all participants, withdrawals will be censored, and odds ratios will be calculated using the inverse probability of censoring weighting instead of Fisher’s exact test. The likelihood of censoring will be estimated for each group using the Kaplan–Meier method by defining censoring as an event. In this case, analysis using Fisher’s exact test will also be performed for reference purposes.

For the incidence of HDP at each specified time point, frequencies, percentages, and a two-tailed 95% confidence interval (CI) for the percentages will be calculated. The CIs will be the exact CIs calculated using the Clopper-Pearson method. Between-group comparisons will be made using Fisher’s least significant difference test, as mentioned above. In Fisher’s least significant difference test, Fisher’s exact test will be used for comparisons between three groups and two groups. For the incidence of HDP, between-group differences and ratios, as well as their two-tailed 95% CIs, will be calculated.

***Secondary endpoint analysis***

Analyses of the secondary endpoints is intended to complement the results of the primary analysis. No adjustments will be made for multiple comparisons in the secondary endpoint analysis. All statistical tests will be based on a two-tailed significance level of 5%. The calculated CIs will be two-tailed 95% CIs.

1) Maternal serum sFlt-1/PlGF ratio as well as sFlt-1 and PlGF levels

Values observed at each specified time point (response variable) will be analyzed using a linear mixed-effects model. The explanatory variables included in the model will be group, number of weeks since randomization, and group by number of weeks since randomization interaction (fixed effects), as well as intersecting for each participant and participant by number of weeks since randomization interaction (random effects). The group will be treated as a categorical variable, whereas the number of weeks since randomization will be treated as a continuous variable. For the values observed at each specified time for each group, point estimates (least squares means), standard errors of the means, and two-tailed 95% CIs will be calculated. Pairwise comparisons among the three groups will be made based on the number of weeks since the randomization interaction.

For the values observed at each specified time, summary statistics (N, mean, standard deviation, minimum, median, and maximum) and a two-tailed 95% CI of the mean will be calculated. The CIs will be calculated according to the t-distribution. A t-test will be used for pairwise comparisons among the three groups. Between-group differences in means and two-tailed 95% CIs will be calculated.

Changes from Visit-3 values will be determined at each specified time. These changes (response variables) will be analyzed using a linear mixed-effects model. Because the changes at visit 3 will be zero in all participants, the explanatory variables included in the model will be determined so that the intercept at visit 3 may be zero. Hence, the explanatory variables will be the number of weeks since randomization and group-by-number of weeks since randomization interaction (fixed effects), as well as the participant's number of weeks since randomization interaction (random effect). The group will be treated as a categorical variable, whereas the number of weeks since randomization will be treated as a continuous variable. For changes at each specified time point in each group, point estimates (least square means), standard errors of the means, and two-tailed 95% CIs will be calculated. Pairwise comparisons among the three groups will be made based on the number of weeks since the randomization interaction.

For changes at each specified time point, summary statistics (N, mean, standard deviation, minimum, median, and maximum) and a two-tailed 95% CI of the mean will be calculated. The CIs will be calculated according to the t-distribution. An analysis of covariance involving baseline as a covariate will be used to make pairwise comparisons among the three groups. Between-group differences in means and two-tailed 95% CIs will be calculated. A paired t-test will be used to compare the values observed at two-time points.

2) Placental weight and umbilical cord blood lipid profile

Data will be summarized in terms of summary statistics (N, mean, SD, minimum, median, and maximum) and two-tailed 95% CI of the means. The CIs will be calculated according to the t-distribution. A t-test will be used for pairwise comparisons among the three groups. Between-group differences in means and two-tailed 95% CIs will be determined.

3) Whether the participant (mother) is proteinuric, develops PE/GH, develops any HDP-related complications (placental abruption; hemolysis, elevated liver enzymes, low platelet count syndrome; or eclampsia), develops severe HDP, and whether pregnancy results in abortion or stillbirth.

Frequencies/percentages and two-tailed 95% CIs for the percentages will be calculated. The CIs will be the exact CIs calculated using the Clopper-Pearson method. Fisher’s exact test will be used for pairwise comparisons between the three groups. Between-group differences, ratios in percentages, and two-tailed 95% CIs will be determined.

4) Neonatal outcomes (birth weight, percentage of SGA neonates, neonatal intensive care unit admission rate, and auditory brainstem response)

Data on categorical variables will be analyzed as follows:

Frequencies/percentages and two-tailed 95% CIs for percentages will be calculated for the percentages. The CIs will be the exact CIs calculated using the Clopper-Pearson method. Fisher’s exact test will be used for pairwise comparisons between the three groups. Between-group differences, ratios in percentages, and two-tailed 95% CI intervals will be determined.

Data on continuous variables will be analyzed as follows:

Data will be summarized in terms of summary statistics (N, mean, SD, minimum, median, and maximum) and two-tailed 95% CI of the means. The CIs will be calculated according to the t-distribution. A t-test will be used for pairwise comparisons among the three groups. Between-group differences in means and two-tailed 95% CIs will be determined.

5) Week at diagnosis of HDP

The cumulative percentage of participants developing HDP will be estimated using the Kaplan–Meier method, and its two-tailed 95% CI will be calculated using the Greenwood method. The median time to event will be estimated using the Kaplan–Meier method, and its two-tailed 95% CI will be calculated using the Brookmeyer-Crowley method. The log-rank test will be used for pairwise comparisons among the three groups. Cox regression analysis will be performed to determine the HRs between the paired groups and their two-tailed 95% (CIs. Data on gestational week and number of weeks since randomization at HDP diagnosis of HDP will be analyzed separately in a similar manner.

Data on the primary and individual secondary endpoints will be subjected to subgroup analysis by gestational week at the time of informed consent (weeks 13–14 vs. 15–16)

***Interim and final analysis***

No interim analyses will be performed. The final analysis will be performed after the completion of the follow-up of all participants and the freezing of all available data. The chief statistician will write and submit the Statistical Analysis Report to the Principal Investigator.

**Data to be collected during visits**

During every visit, the participants will be interviewed about their symptoms and examined by inspection and palpation for physical findings. Participants (mother, fetus, or neonate) will also be monitored for signs of adverse events (defined as any unfavorable or unintended disease or sign thereof [including abnormal laboratory findings] that occur in a participant, whether related to the study). Blood pressure will be measured in the sitting position after rest. Body weight will be measured, and the body mass index will be calculated. The laboratory tests shown in Table 2 will also be performed at the times indicated in the study schedule provided in Additional File 1: Table S1.

In addition, obstetric sonography will be performed to ascertain Biparietal diameter (BPD), abdominal circumference (AC), and femur length (FL) will be measured. The estimated fetal body weight (EFBW) will be calculated using the following formula:

EFBW (g) = 1.07 × BPD (cm)^3^ + 0.3 × AC (cm)^2^ × FL (cm)

Some ultrasonographs are designed to measure the anteroposterior trunk diameter (APTD) and transverse trunk diameter (TTD) instead of the AC. If such an instrument is used, EFBW will be calculated using the following formula:

EFBW (g) = 1.07 × BPD (cm)^3^ + 3.42 × APTD (cm) × TTD (cm) × FL (cm)

Information will also be collected on parturition (gestational week at parturition, date of parturition, placental weight, type of parturition [natural, induced, cesarean section, vacuum, or breech extraction], maternal and/or fetal indication for cesarean section, and live/stillbirth) and neonatal status (sex, birth weight, Apgar score [at 1 minute and 5 minutes after birth], umbilical cord [arterial and venous] blood gas profile [may be omitted if umbilical cord blood cannot be collected as it is left to the fetus (e.g., prematurely born baby) for milking], auditory brainstem response [only if assessed], whether the neonate is admitted to the NICU, and whether the neonate is SGA). Adverse neonatal events will be evaluated and documented.

The placenta will be examined histopathologically to confirm the presence of abnormalities. Finally, adherence to the study treatment (from the first day of treatment until treatment completion), use of concomitant drugs or treatments (from informed consent until the end of follow-up), and development of HDP (throughout the study) will be evaluated. No restrictions will be imposed on concomitant drugs or treatments. Decisions on which drugs or treatments should be used concomitantly will be made by physicians in the context of daily practice.

**Compliance with the Protocol and Modifications to the Protocol**

***Compliance with the protocol***

Investigators and sub-investigators will conduct this study in compliance with this protocol unless this may jeopardize subject’s safety or rights.

***Modifications to the protocol***

1) When making any change (excluding minor changes to the study execution plan) to the study execution plan, the study protocol (including the informed consent document), and other relevant written procedures, the Principal Investigator will receive review and approval from the University of Tokyo Clinical Research Review Board in advance. The Principal Investigator will then report the approved change to the institution's manager according to its established procedures and inform investigators at other participating institutions. When receiving such information, the investigator will report the change to the institution manager or ask for their permission according to a relevant rule of the institution. All investigators/sub-investigators must not implement any change to the study protocol (including the informed consent document) without receiving prior approval from the University of Tokyo Clinical Research Review Board and prior permission from the institution manager.

2) If a protocol change gives rise to a change to the study execution plan (excluding minor changes), the Principal Investigator will receive approval of the change from the University of Tokyo Clinical Research Review Board and then submit a study execution plan change notice via the Japanese Registry of Clinical Trials (jRCT) to the Minister of HLW. All investigators/sub-investigators must not implement any change to the study protocol (including the informed consent document) before the Principal Investigator submits a study execution plan change notice to the Minister of HLW and before the plan change is posted at jRCT.

3) When making a minor change to the study execution plan, the Principal Investigator will notify the University of Tokyo Clinical Research Review Board of the change and then submit a study execution plan change notice via jRCT to the Minister of HLW within 10 days after making the change.

**Study Administration and Organization**

The Principal Investigator is responsible for overseeing and directing the study in collaboration with designated key personnel, including the Data Management Manager, Monitoring Manager, Chief Study Statistician, and Auditing Manager. The coordinating center provides operational support through the Consultation Office, Monitoring Office, and Safety Information Office at the Clinical Research Support Center, The University of Tokyo Hospital. In addition, oversight of safety and efficacy is provided by an independent Data and Safety Monitoring Board. Further information is provided in the trial protocol.

**References**

1. Costantine MM, Cleary K, Hebert MF, Ahmed MS, Brown LM, Ren Z, et al. Safety and pharmacokinetics of pravastatin used for the prevention of preeclampsia in high-risk pregnant women: a pilot randomized controlled trial. Am J Obstet Gynecol. 2016;214(6):720.e1–720.e17. doi: [10.1016/j.ajog.2015.12.038](https://doi.org/10.1016/j.ajog.2015.12.038).
2. Costantine MM, West H, Wisner KL, Caritis S, Clark S, Venkataramanan R, et al. A randomized pilot clinical trial of pravastatin versus placebo in pregnant patients at high risk of preeclampsia. Am J Obstet Gynecol. 2021;225(6):666.e1–666.e15. doi: [10.1016/j.ajog.2021.05.018](https://doi.org/10.1016/j.ajog.2021.05.018).
