# Supplementary material for: Pravastatin for the prevention of recurrent hypertensive disorders of pregnancy: study protocol for a randomized, open-label, parallel-group, three-arm trial
Source: Trials. 2025 Nov 12;26:499. doi: 10.1186/s13063-025-09136-7 (PMC12613540; doi:10.1186/s13063-025-09136-7)
Supplement: Supplementary file 3 — Additional file 3. Statistical Analysis Plan. Statistical analysis plan (SAP) version 1.0. [file 13063_2025_9136_MOESM3_ESM.docx]

**Additional File 3**

Statistical Analysis Plan (SAP)

Version 1.0

Study of Pravastatin for Prevention of
Recurrent Hypertensive Disorders of Pregnancy

|  | Signature |  | Date |  |
| --- | --- | --- | --- | --- |
| Chief Study Statistician  Kosuke Kashiwabara |  |  | (*yyyy*)/(*mm*)/(*dd*) |  |
| Principal Investigator  Keiichi Kumasawa |  |  | (*yyyy*)/(*mm*)/(*dd*) |  |
|  |  |  |  |  |

Revision History

| SAP Ver. | Protocol Ver. | Release/Revision Date | Author | Details |
| --- | --- | --- | --- | --- |
| 1.0 | 5.0 | November 16, 2023 | Kosuke Kashiwabara  Takuya Kawahara  Yosuke Inaba | Initial version |
|  |  |  |  |  |
|  |  |  |  |  |
|  |  |  |  |  |

List of Abbreviations and Terms

The abbreviations and terms used in this SAP are defined as follows:

| Abbreviations | Non-abbreviated terms |
| --- | --- |
| ALP | alkaline phosphatase |
| ALT | alanine aminotransferase |
| AST | aspartate aminotransferase |
| BMI | body mass index |
| CK | creatine kinase |
| CKD | chronic kidney disease |
| CRF | case report form |
| Cre | creatinine |
| E2 | estradiol |
| FAS | full analysis set |
| FSH | follicle-stimulating hormone |
| HDL-C | high-density lipoprotein cholesterol |
| IPCW | inverse probability of censoring weighting |
| LDH | lactate dehydrogenase |
| LDL-C | low-density lipoprotein cholesterol |
| LH | luteinizing hormone |
| NICU | neonatal intensive care unit |
| NSE | neuron specific enolase |
| PE | preeclampsia |
| PPS | per protocol set |
| PT | preferred term |
| PlGF | placental growth factor |
| SAS | safety analysis set |
| SGA | small for gestational age |
| SOC | system organ class |
| T-Bil | total bilirubin |
| T-Chol | total cholesterol |
| TG | triglyceride |
| TSH | thyroid-stimulating hormone |
| sFlt-1 | soluble fms-like tyrosine kinase-1 |

Tabel of Contents

[1. Aim of this SAP 8](#_Toc159493365)

[2. Outline of the Study 8](#_Toc159493366)

[2.1 Study Objectives 8](#_Toc159493367)

[2.2 Subjects (indication studied) 8](#_Toc159493368)

[2.2.1 Inclusion criteria 8](#_Toc159493369)

[2.2.2 Exclusion criteria 8](#_Toc159493370)

[2.3 Study design and outline 9](#_Toc159493371)

[2.4 Schedule of observations/examinations/assessments 10](#_Toc159493372)

[2.5 Study Period 11](#_Toc159493373)

[2.6 Subject enrollment and treatment allocation 11](#_Toc159493374)

[2.7 Planned sample size and its rationales 11](#_Toc159493375)

[3. Study Endpoints 12](#_Toc159493376)

[3.1 Primary endpoint 12](#_Toc159493377)

[3.2 Secondary endpoints 12](#_Toc159493378)

[3.2.1 Safety outcomes 12](#_Toc159493379)

[4. General Rules of Statistical Analyses 13](#_Toc159493380)

[4.1 Summary statistics and how to express values/data 13](#_Toc159493381)

[4.2 Statistical software used 13](#_Toc159493382)

[5. Handling of Data 13](#_Toc159493383)

[6. Analysis Sets 14](#_Toc159493384)

[6.1 Definitions of analysis sets 14](#_Toc159493385)

[6.1.1 Full analysis set 14](#_Toc159493386)

[6.1.2 Full analysis set 2 14](#_Toc159493387)

[6.1.3 Per protocol set 15](#_Toc159493388)

[6.1.4 Safety analysis set 15](#_Toc159493389)

[6.2 Breakdown of analysis sets 15](#_Toc159493390)

[6.2.1 Breakdown of analysis sets 15](#_Toc159493391)

[6.2.2 Reasons for exclusion from analysis sets 15](#_Toc159493392)

[6.3 Subjects discontinued from the study during the study period 15](#_Toc159493393)

[7. Subjects’ Information 16](#_Toc159493394)

[7.1 Distribution of data on baseline variables 16](#_Toc159493395)

[7.1.1 Subjects’ demographic and baseline characteristics 16](#_Toc159493396)

[7.1.2 Pregnancy status 16](#_Toc159493397)

[7.1.3 Obstetric history 16](#_Toc159493398)

[7.1.4 Concomitant and previous illnesses 16](#_Toc159493399)

[7.1.5 Symptoms and clinical signs 16](#_Toc159493400)

[7.1.6 Vital signs 17](#_Toc159493401)

[7.1.7 Weight and body mass index (BMI) 17](#_Toc159493402)

[7.1.8 Hematological examination 17](#_Toc159493403)

[7.1.9 Blood chemistry test (lipid profile) 17](#_Toc159493404)

[7.1.10 Blood chemistry test (hepatic, biliary, and pancreatic functions) 17](#_Toc159493405)

[7.1.11 Blood chemistry test (renal function and muscle health) 17](#_Toc159493406)

[7.1.12 Urinalysis 17](#_Toc159493407)

[7.2 Adherence to study treatment 17](#_Toc159493408)

[7.2.1 Treatment adherence rate 17](#_Toc159493409)

[8. Efficacy Analyses 18](#_Toc159493410)

[8.1 Primary outcome measure (incidence of HDP) 18](#_Toc159493411)

[8.1.1 Primary analysis: Incidence rate and between-group comparisons 18](#_Toc159493412)

[8.1.2 Secondary analysis of the primary outcome measure (incidence of HDP) 19](#_Toc159493413)

[8.2 Analyses of secondary outcome measures 19](#_Toc159493414)

[8.2.1 HDP 19](#_Toc159493415)

[8.2.2 Gestational hypertension (GH) 20](#_Toc159493416)

[8.2.3 Preeclampsia (PE) 20](#_Toc159493417)

[8.2.4 Maternal proteinuria 20](#_Toc159493418)

[8.2.5 HDP-related complications 21](#_Toc159493419)

[8.2.6 Severe HDP 21](#_Toc159493420)

[8.2.7 Abortions and stillbirths 21](#_Toc159493421)

[8.2.8 Maternal serum sFlt-1/PlGF ratio 21](#_Toc159493422)

[8.2.9 Maternal serum sFlt-1 level 22](#_Toc159493423)

[8.2.10 Maternal serum PlGF level 22](#_Toc159493424)

[8.2.11 Placental weight 22](#_Toc159493425)

[8.2.12 Umbilical cord blood lipid profile 23](#_Toc159493426)

[8.2.13 Neonatal information 23](#_Toc159493427)

[8.3 Subgroup analyses 23](#_Toc159493428)

[8.4 Other analyses 23](#_Toc159493429)

[8.4.1 Weight and BMI 23](#_Toc159493430)

[8.4.2 Obstetric sonography 23](#_Toc159493431)

[8.4.3 Parturition information 24](#_Toc159493432)

[9. Safety Analyses 24](#_Toc159493433)

[9.1 Assessment of adverse events 24](#_Toc159493434)

[9.1.1 Adverse events 24](#_Toc159493435)

[9.1.2 Pravastatin-related adverse events 25](#_Toc159493436)

[9.1.3 Aspirin-related adverse events 25](#_Toc159493437)

[9.1.4 Serious adverse events and serious treatment-related adverse events 25](#_Toc159493438)

[9.2 Adverse events occurring to fetuses 26](#_Toc159493439)

[9.3 Adverse events occurring to neonates 26](#_Toc159493440)

[9.4 Abortions and stillbirths 26](#_Toc159493441)

[9.5 Clinical/laboratory values observed over time 27](#_Toc159493442)

[9.5.1 Symptoms and clinical signs 27](#_Toc159493443)

[9.5.2 Vital signs 27](#_Toc159493444)

[9.5.3 Weight and BMI 27](#_Toc159493445)

[9.5.4 Hematological parameters 27](#_Toc159493446)

[9.5.5 Blood biochemical (lipid profile) parameters 27](#_Toc159493447)

[9.5.6 Blood biochemical (hepatic, biliary, and pancreatic function) parameters 27](#_Toc159493448)

[9.5.7 Blood biochemical (renal function and muscular health) parameters 27](#_Toc159493449)

[10. Administrative Structure 27](#_Toc159493450)

# 1. Aim of this SAP

This SAP stipulates the details of statistical analyses of data from the Study of Pravastatin for Prevention of Recurrent Hypertensive Disorders of Pregnancy (hereinafter referred to as the study).

# 2. Outline of the Study

## 2.1 Study Objectives

This study is designed to evaluate the safety and efficacy of pravastatin orally administered for preventing HDP to pregnant women at high risk for developing HDP (high-risk pregnant women) who have a history of HDP. For this purpose, the incidence of HDP will be compared between those treated with pravastatin (5 mg/day or 10 mg/day) and those not treated with the drug.

## 2.2 Subjects (indication studied)

Pregnant women who have developed HDP during their previous pregnancies

### 2.2.1 Inclusion criteria

Those who meet all the following requirements will be considered for admission to the study:

(1) Having a history of HDP during any previous pregnancy

(2) Gestational age between 3w+0d and 16w+6d at informed consent

(3) Chronological age between 18 and 45 years at informed consent

(4) Female sex

(5) Having given written informed consent to the study from her own will prior to enrollment

(6) Able to make outpatient visits as scheduled for the study

### 2.2.2 Exclusion criteria

Those who have any of the following conditions will be excluded from the study:

(1) Diagnosed APS

(2) Chronic hypertension

(3) Multiple pregnancy

(4) Severe obesity defined as body mass index (BMI) ≥30 kg/m^2^ at informed consent

(5) A history of allergy to aspirin, any salicylate, or pravastatin

(6) A history of serious drug allergy (e.g., anaphylactic shock) or any serious adverse drug reaction

(7) Participation in any clinical research (e.g., clinical trial) and receipt of either study drug within 17 weeks prior to giving informed consent

(8) Presence or history of any serious hepatic disorder

(9) Alcoholism

(10) Stage ≥4 chronic kidney disease

(11) Current treatment with any fibrate, immunosuppressant, or nicotinate

(12) Untreated hypothyroidism (excluding latent hypothyroidism)

(13) Any hereditary muscle disease or a family history thereof

(14) A history of drug-induced muscle disorder

(15) Peptic ulcer

(16) Bleeding diathesis (excluding aspirin-induced one)

(17) Aspirin-induced asthma or a history thereof

(18) Any other condition that disqualifies the patient for the study in the opinion of the investigator or any designated sub-investigator

## 2.3 Study design and outline

This will be a confirmatory, open-label, randomized controlled study. The study design is outlined in the following figure:

Pravastatin one 5-mg tablet once daily

Bayaspirin one 100-mg tablet once daily

Pravastatin one 10-mg tablet once daily

Bayaspirin one 100-mg tablet once daily

Gestational week

End of
follow-up

Pravastatin:
end of treatment

Bayaspirin:
end of treatment

Start of treatment

13w 0d – 16w 6d

0 – 3 weeks

13w 0d – 16w 6d

35w 6d

Parturition

4 weeks

Informed consent

Enrollment and randomization

Eligibility check

Bayaspirin one 100-mg tablet once-daily

## 2.4 Schedule of observations/examinations/assessments

| Study event | | | Run-in period | Treatment period | | | | | | | Post-treatment  follow-up period* | |
| --- | --- | --- | --- | --- | --- | --- | --- | --- | --- | --- | --- | --- |
| Visits | | | Visit 1 | Visit 2 | Visit 3 | Visit 4 | Visit 5 | Visit 6 | Visit 7 | Visit 8 | Visit 9 | Visit 10 |
| Timing | | | 0-3 weeks before Visit 2 | Start of treatment | 7 weeks after the start of treatment | 11 weeks after the start of treatment | 15 weeks after the start of treatment | 19 weeks after the start of treatment | 23 weeks after the start of treatment or  at end of treatment | At time of parturition | 1 month after parturition (end of treatment) | 3 months after parturition (end of treatment) |
| Acceptable time window | | |  |  | ± 1 week | ± 1 week | ± 1 week | ± 1 week | ± 1 week |  | ± 1 week | ± 1 week |
| Gestational week | | | 13-16w | 13-16w | 20-23w | 24-27w | 28-31w | 32-35w | 36-39w |  |  |  |
| Informed consent | | | ○ |  |  |  |  |  |  |  |  |  |
| Checking subjects’ demographic  and baseline characteristics | | | ○ |  |  |  |  |  |  |  |  |  |
| Randomization | | |  | ○^f^ |  |  |  |  |  |  |  |  |
| Study treatment  (Bayaspirin) | | |  |  |  |  |  | until 35w+6d |  |  |  |  |
| Study treatment  (Pravastatin) | | |  |  |  |  |  |  |  |  |  |  |
| Symptoms and clinical signs | | | ○ | ○ | ● | ● | ● | ● | ● | ● | ● | ● |
| Monitoring for adverse events | | |  |  |  |  |  |  |  |  |  |  |
| Vital signs | | | ○ | ○ | ● | ● | ● | ● | ● | ● | ● | ● |
| Body weight measurement | | | ○ | ○ | ● | ● | ● | ● | ● |  |  |  |
| Laboratory tests | Hematological examination^a^ | | ○ |  | △ | △ | △ | △ | △ |  | △ | △ |
|  | Blood chemistry test | Lipid profile^b^ | ○ |  | ● |  | ● |  | ● |  |  |  |
|  |  | AST, ALT, ALP,  LDH, T-Bil | ○ |  | ● | △ | △ | △ | △ |  | △ | △ |
|  |  | CK, Cre | ○ |  | △ | △ | △ | △ | △ |  | △ | △ |
|  |  | sFlt-1, PIGF, sFlt-1/PIGF |  |  | ● | ● | ● | ● | ● |  |  |  |
|  | Urinalysis^c^ | | ○ | ○ | ● | ● | ● | ● | ● |  | △ | △ |
|  | Umbilical cord blood test^d^ | |  |  |  |  |  |  |  | ● |  |  |
| Obstetric sonography^e^ | | |  |  | ● | ● | ● | ● | ● |  |  |  |
| Parturition information | | |  |  |  |  |  |  |  | ● |  |  |
| Neonatal information | | |  |  |  |  |  |  |  | ● |  |  |
| Neonatal monitoring for  adverse events | | |  |  |  |  |  |  |  |  |  |  |
| Histopathological examination | | |  |  |  |  |  |  |  | ● |  |  |
| Checking adherence to study treatment | | | g | g |  |  |  |  |  |  |  |  |
| Checking and identifying concomitant drugs/treatments | | |  |  |  |  |  |  |  |  |  |  |

○ Events before the start of study treatment ● Events after the start of study treatment

△ Optional events after hypertension found for the first time after gestational week 20

*: Subjects who develop hypertension for the first time after gestational week 20 will be followed up until 3 months after parturition, while those who do not will be followed up until 1 month after parturition.

🞄 Visit 1 and Visit 2 can be made on the same day.

🞄 Hematological examination and blood chemistry test scheduled at Visit 1 can be omitted if sufficient data have been collected during the previous 4 weeks.

🞄 Visit 3 must be made from gestational week 20 onward.

a: The parameters to be assessed will include white blood cell count, differential neutrophil count (%), differential lymphocyte count (%), red blood cell count, hemoglobin concentration, hematocrit, and platelet count.

b: The parameters to be assessed will include T-Chol, TG, LDL-C, and HDL-C.

c: Quantitative determination of protein in urine (protein/creatinine ratio [mg/mg⋅Cre] using a casual urine sample) or qualitative test for proteinuria will be performed.

d: The parameters to be assessed will include sFlt-1, PlGF, sFlt-1/PlGF, T-Chol, TG, LDL-C, HDL-C, AST, ALT, CK, NSE, TSH, FSH, LH, and E2.

e: The parameters to be assessed will include BPD, AC, and FL.

f: Randomization will be done at Visit 2 (start of treatment). If Visit 1 and Visit 2 are made on different days, randomization can be done on the day before Visit 2 (start of treatment).

g: It will be checked whether the subject is currently receiving oral aspirin.

## 2.5 Study Period

(1) Period of subject enrollment: From May 11, 2023 (date of publishing the execution plan) to April 30, 2025

(2) Period of follow-up: From May 11, 2023 (date of publishing the execution plan) to December 31, 2025

(3) Period of the study: From May 11, 2023 (date of publishing the execution plan) to December 31, 2026

## 2.6 Subject enrollment and treatment allocation

🞄 Allocation: Minimization method

🞄 Randomization factors: Age (≥40 or <40 years) and history of PE (yes or no)

## 2.7 Planned sample size and its rationales

A total of 90 subjects are planned to be randomized to receive pravastatin at 10 mg/day (n=30) or 5 mg/day (n=30) or not to receive pravastatin (n=30).

[Rationales]

The primary hypothesis to be tested in this study is that the study drug (pravastatin) at 10 mg/day or 5 mg/day is superior to non-treatment in preventing HDP in pregnant women at high risk for developing HPD who have a history of this condition. Previous studies evaluating this indication for pravastatin have given the following results: in 2016, Constantine et al. reported that among pregnant women at high risk for developing PE, PE occurred in 4/10 subjects treated with placebo compared with no subjects treated with pravastatin at 10 mg/day. In 2021, Constantine et al. reported that PE occurred in 5/10 subjects treated with placebo compared with 2/10 subjects treated with pravastatin at 20 mg/day. Based on these results, the incidence of HDP in this study is expected to be 10% in the pravastatin 10 mg/day group and 50% in the non-treated group. Assuming that the significance level for the entire study is 5% on a two-tailed basis and that Fisher’s least significant difference test (Fisher’s exact test for 3-group and 2-group comparisons) is used, a sample size of n=27 per group would provide the study with various statistical powers to show the superiority of the active treatment (pravastatin 10 mg/day and/or 5 mg/day) over non-treatment based on various hypotheses concerning incidence of HDP (see the table below).

Table. Statistical powers provided by a sample size of n=27 per group in relation to the hypothetical incidence of HDP

|  | Hypothetical incidence of HDP (%) | | | | Statistical power (%)* | | | |  |
| --- | --- | --- | --- | --- | --- | --- | --- | --- | --- |
|  | Pravastatin 10 mg/day group | Pravastatin 5 mg/day group | Non-treated group | Superiority of pravastatin at 10 mg/day | | Superiority of pravastatin at 5 mg/day | Superiority of pravastatin at either dose level | Superiority of pravastatin at both dose levels | |
| Hypothesis 1 | 10 | 10 | 50 | 86.7 | | 87.0 | 93.9 | 79.8 | |
| Hypothesis 2 | 10 | 10 | 40 | 62.3 | | 61.7 | 74.7 | 49.3 | |
| Hypothesis 3 | 10 | 20 | 50 | 84.8 | | 53.8 | 86.7 | 51.9 | |
| Hypothesis 4 | 10 | 20 | 40 | 59.3 | | 26.1 | 61.7 | 26.1 | |

*Superiority of one over another is defined as follows:

Superiority of pravastatin at 10 mg/day: Significantly lower incidence of HDP in the pravastatin 10 mg/day group versus the non-treated group

Superiority of pravastatin at 5 mg/day: Significantly lower incidence of HDP in the pravastatin 5 mg/day group versus the non-treated group

Superiority of pravastatin at either dose level: Significantly lower incidence of HDP in either the pravastatin 10 mg/day or 5 mg/day group versus the non-treated group

Superiority of pravastatin at both dose levels: Significantly lower incidence of HDP in both the pravastatin 10 mg/day and 5 mg/day groups versus the non-treated group

If pravastatin proves to be effective at either 10 mg/day or 5 mg/day for the prevention of HDP, its clinical use for this new indication is expected. Considering this, the sample size is set at n=27 per group as this will provide the study with sufficient power to show the superiority of pravastatin at either dose level under both Hypothesis 1 (as high efficacy as expected) and Hypothesis 2 (lower efficacy than expected) as shown in the above table. This sample size can also power the study adequately to show the efficacy of pravastatin at 10 mg/day under Hypothesis 3 (lower efficacy than expected only at 5 mg/day) and under Hypothesis 4 (unexpectedly higher or lower incidence of HDP in both the pravastatin 5 mg/day and non-treated groups) (despite a slightly reduced power to show the superiority of pravastatin at 10 mg/day under Hypothesis 4 compared to Hypothesis 2).

Based on these considerations and taking an expected dropout rate of 10% into account, the target sample size is set at n=30 per group.

# 3. Study Endpoints

## 3.1 Primary endpoint

Incidence of HDP

## 3.2 Secondary endpoints

(1) Incidence rates of PE and GH (two major categories of HDP)

(2) Maternal serum sFlt-1/PlGF ratio as well as sFlt-1 and PlGF levels

(3) Whether the subject (mother) is proteinuric

(4) Placental weight

(5) Umbilical cord blood lipid profile

(6) Incidence of HDP-related complications (placental abruption, HELLP syndrome, and eclampsia)

(7) Week at diagnosis of HDP

(8) Incidence of severe HDP

(9) Neonatal outcomes (birth weight, percentage of SGA neonates, NICU admission rate, and auditory brainstem response)

(10) Frequencies and percentages of abortions and stillbirths per pregnancy

### 3.2.1 Safety outcomes

(1) Frequency/percentage of subjects experiencing adverse events

(2) Frequency/percentage of abortions and stillbirths per pregnancy

# 4. General Rules of Statistical Analyses

## 4.1 Summary statistics and how to express values/data

1) Summary statistics for continuous variables will include number, mean, standard deviation, minimum, median, and maximum. For categorical variables, summary statistics will include frequency and percentage.

2) The number of significant digits for means, standard deviations, and medians will be the number of significant digits for individual data plus 1. For example, if the number of significant digits for individual data on a particular variable is at the first decimal place, their mean, standard deviation, and median will be calculated to the second decimal place. The number of significant digits for minimums and maximums of data will be the same as that for the individual data. However, if data clearly have a greater number of digits than the true number of significant figures, such as those on a derived variable, the number of digits that should be displayed will be determined appropriately considering the number of significant digits.

3) Frequency will be expressed as an integer. The percentage will be expressed as a percent and rounded to the first decimal place.

4) Values calculated by analysis, such as least square means and their confidence intervals (CIs), will be rounded to the third decimal place. If this rule clearly ignores the number of significant digits, the number of digits displayed will be changed accordingly.

5) A p-value less than 0.001 will be expressed as “<0.001”. A p-value not less than 0.001 will be rounded to the third decimal place.

## 4.2 Statistical software used

SAS Version 9.4 or a newer version of SAS (SAS Institute Inc.) will be used as the software for analysis, pooling, and graphic representation/tabulation of data from the study.

# 5. Handling of Data

Upon pooling/analysis, data will be handled, in principle, as specified below. If any question arises, it will be discussed and cleared by the Principal Investigator and the Chief Study Statistician. Missing data will not be imputed, in principle. Outliers/abnormal values will be excluded only if they can be clearly explained clinically (e.g., hemolysis). Any specific rule applied to data handling will be specified in Section 7 or a subsequent section of this SAP.

1) Handling of baseline data

Data collected at Visit 1 will be regarded as baseline for all variables assessed at multiple time points.

2) Diagnosis of HDP

Subjects reported to develop either gestational hypertension (GH), or preeclampsia (PE) after gestational week 20 in the HDP page of their case report forms (CRFs) will be diagnosed with HDP.

3) Treatment adherence rate

The treatment adherence rate will be calculated using the following formula:

Treatment adherence rate for pravastatin = (number of days of follow-up – number of days with missed doses of pravastatin)/ number of days of follow-up*

* Number of days of follow-up = date of completing treatment – date of initiating treatment + 1

If the treatment is terminated before parturition, the date of termination will be the date of completing treatment.

4) Whether the subject (mother) is proteinuric

A subject will be diagnosed with proteinuria if:

🞄 the quantitatively determined urinary protein/creatinine ratio is ≥0.3 mg/mg⋅Cre, or

🞄 2 or more successive qualitative tests for proteinuria performed on a single day gives a ≥1+ response.

5) Gestational week

The gestational week on a particular day will be calculated by adding the gestational week at informed consent as specified on the Pregnancy page of the CRF to the number of weeks since the time of informed consent.

6) Obstetric outcome

A subject will be regarded as giving birth if she has a live birth (including a neonatal death) or a stillbirth from gestational week 22 onward.

7) Acceptability of data in relation to the timing of their collection

Only data collected within the acceptable time window specified in Section 2.4 of this SAP will be analyzed. One exception is time-to-event data, which will be assessed regardless of whether the data are collected within the acceptable time window.

# 6. Analysis Sets

## 6.1 Definitions of analysis sets

### 6.1.1 Full analysis set

The Full Analysis Set (FAS) will be composed of those enrolled in this study and treated with at least one dose of the study drug(s) after randomization and who have no major protocol violations (e.g., not giving informed consent and being enrolled outside the period of enrollment).

### 6.1.2 Full analysis set 2

The Full Analysis Set 2 (FAS2) will be composed of FAS subjects who do not develop hypertension before gestational week 20.

### 6.1.3 Per protocol set

The Per Protocol Set (PPS) will be composed of FAS2 subjects who are more compliant with the protocol, i.e., have no significant protocol violations regarding study methodology and concomitant treatments such as:

🞄 Not meeting the inclusion criteria

🞄 Having any of the conditions included in the exclusion criteria

🞄 Being not fully (<80%) adherent to pravastatin treatment

### 6.1.4 Safety analysis set

The safety analysis set will be composed of those enrolled in this study and treated with at least one dose of the study drug(s).

## 6.2 Breakdown of analysis sets

Population to be analyzed: All subjects enrolled in the study

### 6.2.1 Breakdown of analysis sets

To prepare a subject flow diagram, the numbers of subjects in the following populations will be calculated: all subjects enrolled, ineligible subjects, subjects randomized, subjects included in the SAS, subjects excluded from the SAS, subjects included in the FAS, subjects excluded from the FAS, subjects included in the FAS2, subjects excluded from the FAS2, subjects included in the PPS, and subjects excluded from the PPS.

### 6.2.2 Reasons for exclusion from analysis sets

If a subject is excluded from any of the following populations, the reason for exclusion will be summarized: SAS, FAS, FAS2, and PPS.

## 6.3 Subjects discontinued from the study during the study period

Population to be analyzed: All patients enrolled in the study

If a subject is discontinued from the study during the study period, the reason for discontinuation will be summarized.

A subject will be discontinued from the study if/when:

1) She develops hypertension at a gestational age of less than 20 weeks

2) She withdraws consent to the study of her own will

3) She becomes unable to continue participation in the study in the opinion of the investigator/sub-investigator because of worsening of the underlying or concomitant condition or occurrence of any serious adverse event

4) She is found to have any significant noncompliance with the Clinical Trials Act or its Enforcement Regulation or any significant deviation from the protocol (e.g., failure to meet the inclusion criteria or presence of any condition included in the exclusion criteria)

5) She becomes unable to comply with the protocol

6) The entire study has been terminated

7) She becomes unable to continue participating in the study for any other reason in the opinion of the investigator/sub-investigator.

# 7. Subjects’ Information

## 7.1 Distribution of data on baseline variables

Populations to be analyzed: FAS, FAS2, PPS, and SAS

Data on baseline variables will be compared between groups using the chi-squared test (nominal variables), the one-way analysis of variance (ANOVA), or the Kruskal-Wallis test (continuous variables). Data will be considered significant when the two-tailed p-value is less than 0.05.

### 7.1.1 Subjects’ demographic and baseline characteristics

Data on the following variables will be summarized by group in terms of summary statistics and statistically compared between groups: age (years), age (≥40 or <40 years), height (cm), race (Japanese or non-Japanese), smoking status (current smoker, never smoker, or ex-smoker), number of cigarettes smoked per day, and number of years of smoking.

### 7.1.2 Pregnancy status

Data on the following variables will be summarized by group in terms of summary statistics and statistically compared between groups: use of assisted reproductive technology for the current pregnancy (yes or no), gestational age at informed consent (week), and gestational age at informed consent (week 13-14 or 15-16).

### 7.1.3 Obstetric history

Data on the following variables will be summarized by group in terms of summary statistics and statistically compared between groups: number of pregnancies, age at pregnancy (years), outcomes of pregnancies (live birth, stillbirth, abortion, or others), week at parturition, and week at abortion. If a subject has multiple data on a single variable, all the data will be included in the analysis; not the number of subjects, but the number of data will be used as the denominator in calculating a percentage.

### 7.1.4 Concomitant and previous illnesses

Data on the following variables will be summarized by group in terms of summary statistics and statistically compared between groups: a history of PE (yes or no) and any other concomitant or previous illness (yes or no).

### 7.1.5 Symptoms and clinical signs

Data on the following variables will be summarized by group in terms of summary statistics and statistically compared between groups: any symptom (yes or no), complaint of headache (yes or no), complaint of sensation of flashing lights (yes or no), any other symptom to be reported (yes or no), and any clinical sign to be reported (yes or no).

### 7.1.6 Vital signs

Data on the following variables will be summarized by group in terms of summary statistics and statistically compared between groups: systolic blood pressure (derived data; mmHg) and diastolic blood pressure (derived data; mmHg).

### 7.1.7 Weight and body mass index (BMI)

Data on the following variables will be summarized by group in terms of summary statistics and statistically compared between groups: weight (kg) and BMI (kg/m^2^).

### 7.1.8 Hematological examination

Data on the following variables will be summarized by group in terms of summary statistics and statistically compared between groups: white blood cell count (/μL), differential neutrophil count (%), differential lymphocyte count (%), red blood cell count (× 10^4^/μL), hemoglobin concentration (g/dL), hematocrit (%), and platelet count (× 10^4^/μL).

### 7.1.9 Blood chemistry test (lipid profile)

Data on the following variables will be summarized by group in terms of summary statistics and statistically compared between groups: T-Chol (mg/dL), TG (mg/dL), LDL-C (mg/dL), and HDL-C (mg/dL).

### 7.1.10 Blood chemistry test (hepatic, biliary, and pancreatic functions)

Data on the following variables will be summarized by group in terms of summary statistics and statistically compared between groups: AST (U/L), ALT (U/L), ALP (U/L), LDH (U/L), and T-Bil (mg/dL).

### 7.1.11 Blood chemistry test (renal function and muscle health)

Data on the following variables will be summarized by group in terms of summary statistics and statistically compared between groups: CK (U/L) and Cre (mg/dL).

### 7.1.12 Urinalysis

Data on the following variables will be summarized by group in terms of summary statistics and statistically compared between groups: maternal proteinuria (yes or no).

## 7.2 Adherence to study treatment

Populations to be analyzed: FAS, FAS2, PPS, and SAS

### 7.2.1 Treatment adherence rate

Data on treatment adherence rate for pravastatin will be summarized by group in terms of summary statistics.

# 8. Efficacy Analyses

All efficacy endpoints will be analyzed based on the intent-to-treat (ITT) principle; comparisons will be made between groups according to assigned treatments. The FAS will be used as the primary population to be analyzed for the primary endpoint. Analyses of the FAS2 and the PPS will also be performed for reference. If two analysis sets are equal, analysis on the second set will be omitted. For example, if analyses on the FAS and FAS2 are planned and if the two analysis sets are equal, the analysis on the FAS2 will be omitted. All statistical hypothesis tests will be based on a two-tailed significance level of 5%. CIs calculated will be two-tailed 95% CIs.

## 8.1 Primary outcome measure (incidence of HDP)

Populations to be analyzed: FAS, FAS2 and PPS

The primary objective of this study is to show the superiority of pravastatin at 10 mg/day and 5 mg/day over non-treatment in reducing the incidence of HDP in pregnant women at high risk for developing HDP who have a history of HDP.

### 8.1.1 Primary analysis: Incidence rate and between-group comparisons

Assessment will be based on the difference in the incidence of HDP in the 10 mg/day and 5 mg/day groups compared to the non-treated group. A hypothesis test for the 10 mg/day group will use the null hypothesis that the incidence of HDP in the 10 m/day group minus the incidence of HDP in the non-treated group is zero and the alternative hypothesis that the incidence of HDP in the 10 mg/day group minus the incidence of HDP in the non-treated group is not zero. A hypothesis test for the 5 mg/day group will use null and alternative hypotheses created similarly. A hypothesis test for between-dose comparison will use the null hypothesis that the incidence of HDP in the 10 mg/day group minus the incidence of HDP in the 5 mg/kg group is zero and the alternative hypothesis that the incidence of HDP in the 10 mg/day group minus the incidence of HDP in the 5 mg/kg group is not zero.

P-values will be adjusted for multiple comparisons using Fisher’s least significant difference test, where Fisher’s exact test will be used for comparisons among the 3 groups and a comparison between the 2 groups. Each null hypothesis will be rejected only if a significant difference is shown by Fisher’s exact test not only for a comparison between the 2 groups but also for comparisons among the 3 groups.

When the point estimate of the between-group difference in a null hypothesis is a negative value, and the null hypothesis is rejected, it will be concluded that the superiority of the treatment indicated is demonstrated. Since adjustments will be made for multiple comparisons, comparisons that show the superiority of one treatment over the other according to the above-mentioned procedures (not only comparisons of 10 mg/day and 5 mg/day groups with the non-treated group but also a comparison between the 10 mg/day and 5 mg/day groups) can be interpreted as demonstrating the superiority.

For incidence of HDP, frequency, percentage, and two-tailed 95% CI for the percentage will be calculated by group. The CIs will be exact CIs calculated by the Clopper-Pearson method. For paired comparisons between the 10 mg/day and non-treated groups, the 5 mg/day and non-treated groups, and the 10 mg/day and 5 mg/day groups, treatment differences and ratios as well as their two-tailed 95% CIs will be calculated.

Subjects withdrawn from the study after gestational week 20 before developing HPD and before parturition will be regarded as not developing HPD. If such subjects account for more than 10% of all subjects, withdrawals will be censored, and odds ratios will be calculated by using inverse probability of censoring weighting (IPCW) instead of using Fisher’s exact test. The probability of censoring will be estimated for each group using the Kaplan-Meier method by defining censoring as the event. In this case, analysis using Fisher’s exact test will also be performed for reference.

The primary analysis will be performed on the FAS; analyses on other populations will also be performed for reference.

### 8.1.2 Secondary analysis of the primary outcome measure (incidence of HDP)

#### 8.1.2.1 Incidence rates and between-group comparisons at each specified time

For incidence of HDP at each specified time (Visits 3 through 8), frequencies/percentages and two-tailed 95% CIs for the percentages will be calculated by group. The CIs will be exact CIs calculated by the Clopper-Pearson method. Fisher’s least significant difference test will be used for exploratory paired comparisons among the 3 groups.

To compare the incidence of HDP at each specified time between the 10 mg/day and non-treated groups, 5 mg/day and non-treated groups, and the 10 and 5 mg/day groups, treatment differences and ratios as well as their two-tailed 95% CIs will be calculated.

## 8.2 Analyses of secondary outcome measures

Populations to be analyzed: FAS2 and PPS

Analysis of secondary endpoints will aim to provide discussions that complement the results of the primary analysis of the study. No adjustments will be made for multiple comparisons in the analysis of secondary endpoints. The FAS2 will be the primary population to be analyzed, and analysis on the PPS will also be performed for reference. If the FAS2 and the PPS are equal, analysis on the PPS will be omitted. All statistical tests will be based on a two-tailed significance level of 5%. The CIs calculated will be two-tailed 95% CIs.

### 8.2.1 HDP

#### 8.2.1.1 Number of weeks since randomization at diagnosis of HDP

The cumulative percentage of subjects developing HDP in each group will be estimated by the Kaplan-Meier method, and its two-tailed 95% CI will be calculated by the Greenwood method. The median time to event will be estimated by the Kaplan-Meier method, and its two-tailed 95% CI will be calculated by the Brookmeyer-Crowley method. The log-rank test will be used for pairwise comparisons among the 3 groups. Cox regression analysis will be performed to determine the HRs between paired groups (3 pairs of 10 mg/day and non-treated groups, 5 mg/day and non-treated groups, and 10 mg/day and 5 mg/day groups) and their two-tailed 95% CIs.

#### 8.2.1.2 Gestational week at diagnosis of HDP

Similar analyses to those specified in Section 8.2.1.1 will be performed.

### 8.2.2 Gestational hypertension (GH)

#### 8.2.2.1 Incidence rate and between-group comparisons

Data will be analyzed in a similar manner to that specified in Section 8.1.1, except that no adjustments will be made for multiple comparisons.

#### 8.2.2.2 Incidence rates and between-group comparisons at each specified time

Similar analyses to those specified in Section 8.1.2.1 will be performed, except that no adjustments will be made for multiple comparisons.

#### 8.2.2.3 Number of weeks since randomization at diagnosis of GH

Similar analyses to those specified in Section 8.2.1.1 will be performed.

#### 8.2.2.4 Gestational week at diagnosis of GH

Similar analyses to those specified in Section 8.2.1.2 will be performed.

### 8.2.3 Preeclampsia (PE)

#### 8.2.3.1 Incidence rate and between-group comparisons

Data will be analyzed in a similar manner to that specified in Section 8.1.1, except that no adjustments will be made for multiple comparisons.

#### 8.2.3.2 Incidence rate and between-group comparisons at each specified time

Similar analyses to those specified in Section 8.1.2.1 will be performed, except that no adjustments will be made for multiple comparisons.

#### 8.2.3.3 Number of weeks since randomization at diagnosis of PE

Similar analyses to those specified in Section 8.2.1.1 will be performed.

#### 8.2.3.4 Gestational week at diagnosis of PE

Similar analyses to those specified in Section 8.2.1.2 will be performed.

### 8.2.4 Maternal proteinuria

#### 8.2.4.1 Incidence rate and between-group comparisons

Frequencies/percentages and two-tailed 95% CIs for the percentages will be calculated by group. The CIs will be exact CIs calculated by the Clopper-Pearson method. Fisher’s exact test will be used for pairwise comparisons among the 3 groups. Between-group differences and ratios in percentages, as well as their two-tailed 95% CIs, will be determined.

#### 8.2.4.2 Incidence rate and between-group comparisons at each specified time

Frequencies/percentages and two-tailed 95% CIs for the percentages will be calculated by group. The CIs will be exact CIs calculated by the Clopper-Pearson method. Fisher’s exact test will be used for pairwise comparisons among the 3 groups. Between-group differences and ratios in percentages, as well as their two-tailed 95% CIs, will be determined.

### 8.2.5 HDP-related complications

#### 8.2.5.1 Incidence rate and between-group comparisons

For any HDP-related complication (placental abruption, HELLP syndrome, or eclampsia), frequencies/percentages and two-tailed 95% CIs for the percentages will be calculated by group. The CIs will be exact CIs calculated by the Clopper-Pearson method. Fisher’s exact test will be used for pairwise comparisons among the 3 groups. Between-group differences and ratios in percentages, as well as their two-tailed 95% CIs, will be determined.

### 8.2.6 Severe HDP

#### 8.2.6.1 Incidence rate and between-group comparisons

Frequencies/percentages and two-tailed 95% CIs for the percentages will be calculated by group. The CIs will be exact CIs calculated by the Clopper-Pearson method. Fisher’s exact test will be used for pairwise comparisons among the 3 groups. Between-group differences and ratios in percentages, as well as their two-tailed 95% CIs, will be determined.

### 8.2.7 Abortions and stillbirths

#### 8.2.7.1 Incidence rate and between-group comparisons

Frequencies/percentages and two-tailed 95% CIs for the percentages will be calculated by group. The CIs will be exact CIs calculated by the Clopper-Pearson method. Fisher’s exact test will be used for pairwise comparisons among the 3 groups. Between-group differences and ratios in percentages, as well as their two-tailed 95% CIs, will be determined.

### 8.2.8 Maternal serum sFlt-1/PlGF ratio

#### 8.2.8.1 Summaries and between-group comparisons of the values observed at each specified time

Data for each group will be summarized in terms of summary statistics and two-tailed 95% CI of the mean. The CI will be calculated according to t-distribution. The t-test will be used for pairwise comparisons among the 3 groups. Between-group differences in means and their two-tailed 95% CIs will be calculated.

#### 8.2.8.2 Values observed over time

For each group, mean values observed over time will be displayed on a graph together with their two-tailed 95% CIs.

Individual subject values observed over time will be displayed on a graph for each group.

#### 8.2.8.3 Between-group comparisons of values observed across the treatment period

Values observed at each specified time (response variable) will be analyzed using a linear mix-effects model. Explanatory variables included in the model will be group, number of weeks since randomization, and group by number of weeks since randomization interaction (fixed effects) as well as the intercept for each subject and subject by number of weeks since randomization interaction (random effects). Group will be handled as a categorical variable, while number of weeks since randomization will be handled as a continuous variable. For values observed at each specified time for each group, point estimates (least square means), standard errors of the means, and two-tailed 95% CIs will be calculated. Pairwise comparisons among the 3 groups will be made based on the group by number of weeks since randomization interaction.

#### 8.2.8.4 Summaries and between-group comparisons of changes from Visit 3 at each specified time

Changes from Visit-3 values at each specified time will be summarized for each group in terms of summary statistics and two-tailed 95% CI of the mean. The CIs will be calculated according to t-distribution. The analysis of covariance (ANCOVA) involving baseline as a covariate will be used for pairwise comparisons among the 3 groups. Between-group differences in means and their two-tailed 95% CIs will be calculated. The paired t-test will be used to compare values observed at two time points.

#### 8.2.8.5 Changes from Visit 3 over time

For each group, mean changes from Visit-3 over time will be displayed on a graph together with their two-tailed 95% CIs.

Individual subject data obtained over time will be displayed on a graph for each group.

#### 8.2.8.6 Between-group comparisons of changes from Visit 3 across the treatment period

Changes from Visit-3 values (response variable) will be analyzed using a linear mix-effects model. Since the changes at Visit 3 will be zero in all subjects, explanatory variables included in the model will be determined so that the intercept at Visit 3 may be zero. Hence, the explanatory variables will be number of weeks since randomization and group by number of weeks since randomization interaction (fixed effects) as well as subject by number of weeks since randomization interaction (random effect). Group will be handled as a categorical variable, while number of weeks since randomization will be handled as a continuous variable. For changes at each specified time for each group, point estimates (least square means), standard errors of the means, and two-tailed 95% CIs will be calculated. Pairwise comparisons among the 3 groups will be made based on the group by number of weeks since randomization interaction.

### 8.2.9 Maternal serum sFlt-1 level

Data will be analyzed in a similar manner to that specified in Section 8.2.8.

### 8.2.10 Maternal serum PlGF level

Data will be analyzed in a similar manner to that specified in Section 8.2.8.

### 8.2.11 Placental weight

#### 8.2.11.1 Summaries and between-group comparisons of values observed

Data will be summarized for each group in terms of summary statistics and two-tailed 95% CI of the mean. CIs will be calculated according to t-distribution. The t-test will be used for pairwise comparisons among the 3 groups. Between-group differences in means and their two-tailed 95% CIs will be determined.

### 8.2.12 Umbilical cord blood lipid profile

#### 8.2.12.1 Summaries and between-group comparisons of values observed

Data on umbilical cord blood laboratory parameters (T-Chol, TG, LDL-C, HDL-C, AST, ALT, CK, NSE, TSH, FSH, LH, and E2) will be summarized for each group in terms of summary statistics and two-tailed 95% CIs of the means. CIs will be calculated according to t-distribution. The t-test will be used for pairwise comparisons among the 3 groups. Between-group differences in means and their two-tailed 95% CIs will be determined.

### 8.2.13 Neonatal information

#### 8.2.13.1 Summaries and between-group comparisons of values observed

Data on categorical variables (sex [male or female], NICU admission [yes or no], being judged as SGA (yes or no), auditory brainstem response [passed or requiring retest], umbilical cord (arterial) blood gas profile [abnormal or normal], and umbilical cord (venous) blood gas profile [abnormal or normal]) will be analyzed as follows:

Frequencies/percentages and two-tailed 95% CIs for percentages will be calculated for each group. The CIs will be exact CIs calculated by the Clopper-Pearson method. Fisher’s exact test will be used for pairwise comparisons among the 3 groups. Between-group differences and ratios in percentages as well as their two-tailed 95% CIs will be determined.

Data on continuous variables (birth weight as well as Apgar score at 1 minute and 5 minutes after birth) will be analyzed as follows:

Data will be summarized for each group in terms of summary statistics and two-tailed 95% CIs of the means. CIs will be calculated according to t-distribution. The t-test will be used for pairwise comparisons among the 3 groups. Between-group differences in means and their two-tailed 95% CIs will be determined.

## 8.3 Subgroup analyses

Data on the subjects’ demographic and baseline characteristics, the primary endpoint, and individual secondary endpoints will be subjected to subgroup analysis by:

Subgroup 1: Subjects giving informed consent at gestational week 13-14

Subgroup 2: Subjects giving informed consent at gestational week 15-16

## 8.4 Other analyses

### 8.4.1 Weight and BMI

#### 8.4.1.1 Summaries of values observed at each specified time

For the following parameters, values observed at each specified time will be summarized for each group in terms of summary statistics: Body weight (kg) and BMI (kg/m^2^).

### 8.4.2 Obstetric sonography

#### 8.4.2.1 Summaries of values observed at each specified time

For the following parameters, values observed at each specified time will be summarized for each group in terms of summary statistics: Biparietal diameter (cm), abdominal circumference (cm), antero-postero trunk diameter (cm), transverse trunk diameter (cm), femur length (cm), and estimated fetal body weight (g).

### 8.4.3 Parturition information

#### 8.4.3.1 Summaries of values observed

For the following parameters, values observed will be summarized for each group in terms of summary statistics: gestational week at parturition, form of parturition (natural, induced, cesarean section, vacuum, or breech extraction), live or still birth, giving birth (yes or no), maternal and/or fetal indication for cesarean section (yes or no), and histopathological findings of placenta (abnormal or normal)

# 9. Safety Analyses

Populations to be analyzed: SAS

Between-group comparisons will be made according to actual treatments received. Safety analyses will be performed on the safety analysis set. All statistical tests will be based on a two-tailed significance level of 5%. CIs calculated will be two-tailed 95% CIs.

## 9.1 Assessment of adverse events

Population to be analyzed: SAS

Adverse events will be coded using the MedDRA/J System Organ Classes (SOCs) and Preferred Terms (PTs). The SOCs used will be primary SOCs.

### 9.1.1 Adverse events

#### 9.1.1.1 Percentage of subjects experiencing adverse events (by SOC and PT)

For each group, the total number of episodes, the number of episodes by SOC/PT, the total number of subjects with any adverse event, and the number/percentage of subjects with adverse events by SOC/PT will be calculated. Fisher’s exact test will be used for pairwise comparisons among the 3 groups (with the level of significance being twice the smaller one-tailed significance level). Fisher’s exact test will also be used for comparisons between the combined pravastatin (10 mg/day plus 5 mg/day) group and the non-treated group.

#### 9.1.1.2 Percentage of subjects experiencing adverse events (by SOC, PT, and grade)

If any moderate or severe adverse event occurs, the total number of episodes by grade, the number of episodes by SOC/PT by grade, the total number of subjects with any adverse event by grade, and the number/percentage of subjects with adverse events by SOC/PT by grade will be calculated for each group. The Cochran-Mantel-Haenszel test (correlation statistic) will be used for pairwise comparisons among the 3 groups and for comparisons between the combined pravastatin (10 mg/day plus 5 mg/day) group and the non-treated group.

### 9.1.2 Pravastatin-related adverse events

#### 9.1.2.1 Percentage of subjects experiencing pravastatin-related adverse events (by SOC and PT)

Similar analyses to those specified in Section 9.1.1.1 will be performed.

#### 9.1.2.2 Percentage of subjects experiencing pravastatin-related adverse events (by SOC, PT, and grade)

Similar analyses to those specified in Section 9.1.1.2 will be performed.

### 9.1.3 Aspirin-related adverse events

#### 9.1.3.1 Percentage of subjects experiencing aspirin-related adverse events (by SOT and PT)

Similar analyses to those specified in Section 9.1.1.1 will be performed.

#### 9.1.3.2 Percentage of subjects experiencing aspirin-related adverse events (by SOC, PT, and grade)

Similar analyses to those specified in Section 9.1.1.2 will be performed.

### 9.1.4 Serious adverse events and serious treatment-related adverse events

#### 9.1.4.1 Percentage of subjects experiencing serious adverse events (by SOC and PT)

Similar analyses to those specified in Section 9.1.1.1 will be performed.

#### 9.1.4.2 Percentage of subjects experiencing serious pravastatin-related adverse events (by SOC and PT)

Similar analyses to those specified in Section 9.1.1.1 will be performed.

#### 9.1.4.3 Percentage of subjects experiencing serious aspirin-related adverse events (by SOC and PT)

Similar analyses to those specified in Section 9.1.1.1 will be performed.

#### 9.1.4.4 Line listing of adverse events

Adverse events will be listed together with the information presented below. In the list, adverse events will be sorted according to group, subject ID, and timing of onset.

🞄 Group

🞄 Subject ID

🞄 Date of giving informed consent

🞄 Participating institution

🞄 Age

🞄 Weight

🞄 Description of the event

🞄 SOC

🞄 PT

🞄 Duration of the event (number of days from onset until confirmation of outcome)

🞄 Grade (mild, moderate or severe)

🞄 Seriousness (see below for the definition of a serious adverse event)

🞄 Outcome (resolved, improved, not recovered, recovered with sequelae, died, or unknown)

🞄 Date of confirming the outcome

🞄 Causal relationship with aspirin (related or unrelated)

🞄 Causal relationship with pravastatin (related or unrelated)

🞄 Date of onset

🞄 Timing of onset (number of days since randomization)

🞄 Measure (aspirin) (permanently discontinued, dose reduced, suspended, unchanged, not applicable, or unknown)

🞄 Measure (pravastatin) (permanently discontinued, dose reduced, suspended, unchanged, not applicable, or unknown)

🞄 Measure (with or without concomitant drugs)

🞄 Measure (with or without concomitant treatments)

🞄 Measure (with or without any other measure)

🞄 Measure (specify any other measures)

An adverse event will be judged as serious if it

1) Results in death

2) Is life-threatening

3) Requires hospitalization or prolonged hospitalization at a clinic or hospital for therapeutic purposes

4) Results in disability

5) May result in disability

6) Is as serious as the above-listed outcomes

7) Is a congenital anomaly or birth defect

## 9.2 Adverse events occurring to fetuses

Similar analyses to those specified in Section 9.1 will be performed.

## 9.3 Adverse events occurring to neonates

Similar analyses to those specified in Section 9.1 will be performed.

## 9.4 Abortions and stillbirths

For each group, number/percentage of pregnancies resulting in abortions or stillbirths and two-tailed 95% CIs for the percentages will be calculated. The CIs will be exact CIs calculated according to the binomial distribution. Fisher’s exact test will be used for pairwise comparisons among the 3 groups. Between-group differences and ratios in percentages as well as their two-tailed 95% CIs will be determined.

## 9.5 Clinical/laboratory values observed over time

For the parameters listed in the following sections, values observed at each specified time will be summarized for each group in terms of summary statistics.

For vital signs, weight, BMI, hematological parameters, and blood biochemical parameters, mean values observed over time will be displayed on a graph for each group together with their two-tailed 95% CIs. Individual subject data obtained over time will be displayed on a graph for each group.

### 9.5.1 Symptoms and clinical signs

Any symptom (yes or no), complaint of headache (yes or no), complaint of sensation of flashing lights (yes or no), any other symptom to be reported (yes or no), and any clinical sign to be reported (yes or no).

### 9.5.2 Vital signs

Systolic blood pressure (derived data; mmHg) and diastolic blood pressure (derived data; mmHg)

### 9.5.3 Weight and BMI

Weight (kg) and BMI (kg/m^2^)

### 9.5.4 Hematological parameters

White blood cell count (/μL), differential neutrophil count (%), differential lymphocyte count (%), red blood cell count (× 10^4^/μL), hemoglobin concentration (g/dL), hematocrit (%), and platelet count (× 10^4^/μL)

### 9.5.5 Blood biochemical (lipid profile) parameters

T-Chol (mg/dL), TG (mg/dL), LDL-C (mg/dL), and HDL-C (mg/dL)

### 9.5.6 Blood biochemical (hepatic, biliary, and pancreatic function) parameters

AST (U/L), ALT (U/L), ALP (U/L), LDH (U/L), and T-Bil (mg/dL)

### 9.5.7 Blood biochemical (renal function and muscular health) parameters

CK (U/L) and Cre (mg/dL)

# 10. Administrative Structure

Chief Study Statistician

Kosuke Kashiwabara

Director, Data Science Office, Investigator-Initiated Clinical Trials Promotion Department,
Clinical Research Promotion Center, The University of Tokyo Hospital

Working statisticians

Takuya Kawahara and Yosuke Inaba

Biostatistics Unit, Data Science Office, Investigator-Initiated Clinical Trials Promotion Department, Clinical Research Promotion Center, The University of Tokyo Hospital
